# *In vitro* activity of *Camellia sinensis* (green tea) against trophozoites and cysts of *Acanthamoeba castellanii*

**DOI:** 10.1016/j.ijpddr.2020.05.001

**Published:** 2020-06-02

**Authors:** Lenu B. Fakae, Carl W. Stevenson, Xing-Quan Zhu, Hany M. Elsheikha

**Affiliations:** a School of Veterinary Medicine and Science, University of Nottingham, Sutton Bonington Campus, Loughborough, LE12 5RD, UK; b School of Biosciences, University of Nottingham, Sutton Bonington Campus, Loughborough, LE12 5RD, UK; c Rivers State University, Nkpolu - Oroworukwo P.M.B 5080, Port Harcourt, Rivers State, Nigeria; d State Key Laboratory of Veterinary Etiological Biology, Key Laboratory of Veterinary Parasitology of Gansu Province, Lanzhou Veterinary Research Institute, Chinese Academy of Agricultural Sciences, Lanzhou, 730046, China

**Keywords:** *Acanthamoeba castellanii*, *Camellia sinensis*, Green tea, Anti-acanthamoebic activity, Cyst, Trophozoite

## Abstract

The effect of *Camellia sinensis* (green tea) on the growth of *Acanthamoeba castellanii* trophozoites was examined using a microplate based-Sulforhodamine B (SRB) assay. *C. sinensis* hot and cold brews at 75% and 100% concentrations significantly inhibited the growth of trophozoites. We also examined the structural alterations in *C. sinensis*-treated trophozoites using transmission electron microscopy (TEM) and scanning electron microscopy (SEM). This analysis showed that *C. sinensis* compromised the cell membrane integrity and caused progressive destruction of trophozoites. *C. sinensis* also significantly inhibited the parasite's ability to form cysts in a dose-dependent manner and reduced the rate of excystation from cysts to trophozoites. *C. sinensis* exhibited low cytotoxic effects on primary corneal stromal cells. However, cytotoxicity was more pronounced in SV40-immortalized corneal epithelial cells. Chromatographic analysis showed that both hot and cold *C. sinensis* brews contained the same number and type of chemical compounds. This work demonstrated that *C. sinensis* has anti-acanthamoebic activity against trophozoite and cystic forms of *A. castellanii*. Further studies are warranted to identify the exact substances in *C. sinensis* that have the most potent anti-acanthamoebic effect.

## Highlights

• *Camellia sinensis* had amoebicidal activity against *Acanthamoeba castellanii* trophozoites.
• *C. sinensis* significantly inhibited the differentiation of trophozoites to cysts in a dose-dependent manner.
• Transient exposure to *C. sinensis* did not significantly inhibit the trophozoites' growth.
• *C. sinensis* was less toxic to primary corneal stromal cells compared to SV40-immortalized corneal epithelial cells.
• High concentrations of *C. sinensis* reduced the excystation of *A. castellanii* cysts.

## Graphical abstract

**Figure:**
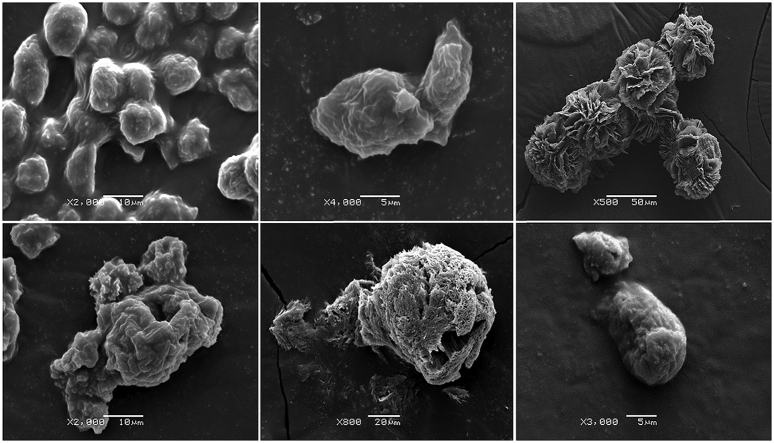

## 1 Introduction

Free-living amoebae are ubiquitously distributed in the environment and can be found in soil and water. One such organism is *Acanthamoeba castellanii*, which can cause serious infections, such as Granulomatous Amoebic Encephalitis (GAE) and *Acanthamoeba* Keratitis (AK) (Martinez and Visvesvara, 1997; Chomicz et al., 2010). The treatment for *A. castellanii* infection has been a challenge, which is currently being managed with drug combinations. Available therapies are lengthy and without a sustained effect, and their withdrawal allows for excystation of encysted parasites and a relapse of infection. The most routinely used chemotherapeutic agents, individually or in combination, are biguanides (e.g. polyhexamethylene [PHMB] or chlorhexidine gluconate [CHX]) and diamidines (propamidine isethionate and hexamidine). These anti-acanthamoebic drugs act by compromising the plasma membrane (biguanides) or interfering with the DNA and protein synthesis (diamidines) of the amoeba (Siddiqui et al., 2016; Kaya et al., 2019). However, they do not completely eradicate the parasite (Gooi et al., 2008), either due to delayed treatment or resistance of the parasite to the treatment agent(s). Other therapies include the use of CHX in combination with diamidines and neomycin (Siddiqui et al., 2016), a combination of anti-infective agents such as trimethoprim/sulfamethoxazole (Aichelburg et al., 2008), ketoconazole (Singhal et al., 2001), dicloxacillin, ciprofloxacin, voriconazole, amphotericin B (Walia et al., 2007), neomycin, metronidazole (Sun et al., 2006), fluconazole, rifampin, pentamidine and macrolide (Visvesvara et al., 2007; Maritschnegg et al., 2011). However, while effective against the trophozoites, these compounds are not highly effective against the cysts, which are made resistant by their cellulose-based double walls. Additionally, side effects and cytotoxicity are a major concern with the prolonged antiacanthamoebic treatment regimens, which can cause damage of the host cells, with a possibility of re-infections (Niyyati et al., 2010; Visvesvara, 2010).

Given the challenges associated with the current treatment protocols, alternative approaches have been suggested in an effort to improve the treatment efficacy (Visvesvara et al., 2007; Gooi et al., 2008; Visvesvara, 2010; Maritschnegg et al., 2011; Kaya et al., 2019). Also, researchers have been delving into natural sources that might have therapeutic potential for treatment of *A. castellanii* infection. These studies have shown anti-acanthamoebic activities of various plants *in vitro* and *in vivo* (Hadas et al., 2017, Hadas et al., 2017; Mahboob et al., 2017; Saoudi et al., 2017; Sifaoui et al., 2017; Dodangeh et al., 2018; Kaynak et al., 2018; Kaya et al., 2019). For example, the methanolic extract of fenugreek (*Trigonella foenum-graecum*) showed amoebicidal activity against *Acanthamoeba* cysts (Kaya et al., 2019) and its seeds exhibited trophicidal and cysticidal activities (Dodangeh et al., 2018). *Ornithogalum sigmoideum* leaves, flowers and stem also exhibited trophicidal and cysticidal effects (Kaynak et al., 2018). Additionally, chloroformic fraction of *Ziziphus vulgaris* (Dodangeh et al., 2017), tea tree oil (Hadas et al., 2017) and extracts of *Centaurea bella, Centaurea daghestanica, Rhaponticum pulchrum,* and *Tanacetum vulgare* (Hadas et al., 2017) exhibited trophicidal and cysticidal effects. Other studies, including the use of Tunisian *Thymus capitatus* essential oil (Saoudi et al., 2017), *Pericampylus glaucus* (Mahboob et al., 2017), and olive leaf extracts (Sifaoui et al., 2013, 2017), all showed anti-acanthamoebic activity.

Green tea (*Camellia sinensis*) contains bioactive compounds, such as flavanols, flavandiols, flavonoids, and phenolic acids, which account for 30% of the dry weight of green tea leaves (Namal Senanayake, 2013; Zhong et al., 2014). It also contains catechins, including (−)-epicatechin (EC), (−)-epicatechin-3-gallate (ECG), (−)-epigallocatechin (EGC), and (−)-epigallocatechin-3-gallate (EGCG) (Mukhtar and Ahmad, 2000). The latter is the most abundant component of *C. sinensis* (Benelli et al., 2002). Catechins have potent anti-oxidant and anti-viral properties, which can be useful for disease prevention amongst other health benefits (Srichairatanakool et al., 2006; Sanlier et al., 2018; Katada et al., 2019; Yang et al., 2019, 2019b). *C. sinensis* also contains caffeine, which is its primary alkaloid and a known CNS stimulant (Perva-Uzunalic et al., 2006), and a host of amino acids, sterols and vitamins. Previous studies have focused on the extraction of polyphenols of *C. sinensis* (Perva-Uzunalic et al., 2006; Namal Senanayake, 2013; Monsanto et al., 2014) and testing *C. sinensis* extracts for potential anti-oxidant, anti-viral, and anti-tumour properties (Mukhtar and Ahmad, 2000; Adhami and Mukhtar, 2007; Dai and Mumper, 2010; Feitelson et al., 2015; Yang et al., 2019). However, the amoebicidal activity of *C. sinensis* extracts remains unknown.

Considering the need for more potent medications against *A. castellanii* infection and given the potential anti-infective properties of *C. sinensis*, the present study was performed to investigate the anti-acanthamoebic activity of hot and cold brews of *C. sinensis* against the trophozoite and cystic stages of *A. castellanii*. Also, the cytotoxicity of *C. sinensis* brews was evaluated on two types of cultured human corneal cells.

## 2 Materials and methods

### 2.1 Parasite culture

*Acanthamoeba castellanii* strain of T4 genotype (American Type Culture Collection; ATCC 30011) was maintained in 20 ml of proteose-peptone-yeast-glucose (PYG) medium [proteose-peptone 0.75% (w/v), yeast extract 0.75% (w/v) and glucose 1.5% (w/v)] in T75 Nunclon cell culture flasks (Thermo Scientific™) at 25 °C in a humidified Stuart™ SI30H Hybridization bench top oven/shaker (Thermo Scientific™) without rocking (Khan et al., 2000). The culture medium was refreshed ~15 h before the commencement of each experiment to ensure that the majority of the culture is composed of vegetative trophozoites.

### 2.2 Preparation of the solutions

Sterile *C. sinensis* brew was prepared in two forms; hot and cold brews. The hot brew was prepared by heating 550 ml of distilled water in a loosely capped glass bottle (Fisher Scientific, UK) in the microwave for 5 min at high power. The volume was adjusted to 500 ml and 5 gm of *C. sinensis* leaves were added. This mixture was shaken and left to stand for 10 min, then filtered using a 500 ml 0.22 µm Stericup® vacuum filtration unit (Merck, UK). The cold brew was prepared by weighing 5 gm of *C. sinensis* leaves into a glass bottle containing 500 ml of distilled water. The mixture was incubated at 4 °C for 24 h and filtered as described for the hot brew.

*C. sinensis*-PYG medium was prepared by dissolving 10 gm glucose monohydrate, (Sigma-Aldrich, France), 7.5 gm of yeast extract (Sigma-Aldrich, France) and 7.5 gm of proteose-peptone (Sigma-Aldrich, Spain) in 500 ml of already prepared sterile *C. sinensis* brew. This was done to ensure that the constituents of PYG media are also available in the *C. sinensis*-PYG media. This suspension was filtered as described above and stored in a glass bottle at 4 °C. To make dilutions of *C. sinensis*-PYG medium, PYG medium was used as a diluent to prepare lower concentrations of *C. sinensis*-PYG medium (i.e. 25, 50, 75%). As for the cytotoxicity assays, sterile *C. sinensis* brew was diluted with the respective corneal cell media (v/v) to achieve the concentration needed.

*C. sinensis* encystation medium was prepared by dissolving 10 gm of glucose monohydrate, 0.48 gm of magnesium chloride (Sigma-Aldrich) and one-fifth of a phosphate-buffered saline (PBS) tablet (Gibco®, Life Technologies, UK) in 100 ml of already prepared sterile *C. sinensis* cold brew. This suspension was filtered into a sterile bottle using a 0.45 µm' Millipore syringe filter (MILLEX®-HA, Ireland). The standardized encystation medium (negative control) was prepared as *C. sinensis* encystation medium, but by using distilled water as a solvent instead of *C. sinensis* brew. The positive control included standardized encystation medium supplemented with 5% phenyl-methanesulfonyl fluoride solution (PMSF).

### 2.3 Cytotoxicity of *C. sinensis* using SRB assay

Corneal stromal cells (CSCs) and SV40-immortalized human corneal epithelial cells (iHCECs) were cultured in M199 medium and Epilife growth medium, respectively. Cell cultures were incubated in a humidified atmosphere of 5% CO_2_ at 37 °C. The cell cultures from each cell type were seeded in 96-well microplates at a density of 5 × 10^3^ cells/well in 100 μl of the respective medium. After 48 h of incubation, the media were removed using an electronic pipette (Gilson), and fresh media containing various concentrations (25%, 50%, 75% and 100%) of cold or hot *C. sinensis* brew were added and incubated for 3, 24, 48 and 72 h. Parallel control wells contained cells with the respective medium only and without any exposure to *C. sinensis* brew. At each time point of incubation, cell proliferation was determined he using the Sulforhodamine B (SRB) assay as described previously (Vichai and Kirtikara, 2006). SRB is a pink aminoxanthene dye that binds to the cellular protein under acidic conditions and dissociates under basic conditions. A spectrophotometer is used to quantify the optical density (OD) of the wells after being treated with the dissociating agent tris-(hydroxylmethyl)-aminomethane. The recorded OD of the supernatant represents the spectrophotometric quantification of the protein concentration of the cells, which is directly proportional to the number of cells (e.g. increased OD correlates with increased protein content, which reflect an increase in the number of cells). Briefly, 25 μl of chilled 10% trichloroacetic acid (10% w/v) (TCA, Fisher, Belgium) were added per well and the plates were incubated for 1 h at 4 °C. The supernatant was discarded, and the plates were washed gently with distilled water three times to remove the TCA and PYG medium, and were subsequently allowed to dry at ambient temperature. Then, 25 μl of SRB solution (0.05% SRB dye dissolved in 1% acetic acid in water) was added per well and incubated for 15 min at ambient temperature protected from light. Excess unbound SRB was removed by gently washing the plates with 1% acetic acid. After the plates were air-dried, 150 μl of 10 mM base solution (tris-(hydroxylmethyl)-aminomethane, pH 10.5) was added per well in order to solubilize the trophozoite protein-bound dye. The plates were subsequently placed on an oscillation rocker (Stuart, UK) for 7 min to achieve a homogenous suspension of the dye in the well's supernatant. The OD of each well was determined by measuring the colour absorbance spectrophotometrically at wavelengths of 450, 492 and 630 nm using an L-T 4000 microplate reader (Labtech, UK). The OD values of each concentration were compared to the other concentrations and to the control at each of the examined time points (i.e. 3, 24, 48 and 72 h).

### 2.4 Cytotoxicity assessment using acridine orange (AO) microplate-based assay

Cells were seeded in 96-well microplates at the same density stated above. At 6, 24, 48 and 72 h post treatment with *C. sinensis* brew, the culture medium was aspirated from each well and the cells were fixed with 30 μl of methanol for 15 min. After fixation, the wells were gently washed twice with distilled water a using multi-channel electronic pipette (Gilson, France). The wells were subsequently incubated with 30 μl of acridine orange dye for 10 min and washed thrice with distilled water. Then, 100 μl of PBS was added to each well and fluorometric quantification of RNA and DNA concentration of the treated cells was done using a Varioskan Flash plate reader (Thermo Scientific, Finland). The excitation/emission was set at 460/650 (nm) for ribonucleic acid (RNA) and 500/526 (nm) for deoxyribonucleic acid (DNA).

### 2.5 Optimization of seeding density

Here, we determined the optimal seeding density of *A. castellanii* trophozoites that were used in testing the anti-acanthamoebic activity of *C. sinensis*. Briefly, *A. castellanii* trophozoites at various numbers (1 × 10^3^, 2.5 × 10^3^, 5 × 10^3^, 7.5 × 10^3^ and 10 × 10^3^) were seeded in a volume of 100 μl PYG medium/well of 96-well plates. Control wells included only 100 μl PYG medium. The culture plates were checked under an inverted microscope (CETI, UK) to ensure the presence of trophozoites, and incubated in a Stuart oven at 25 °C. After 24, 48 and 72 h, the plates were fixed and stained using the SRB assay as described above and strictly according to the protocol adapted for *A. castellanii* by Ortega-Rivas et al. (2016). The OD of each well was determined by measuring the colour absorbance spectrophotometrically at wavelengths of 450, 492 and 630 nm using an L-T 4000 microplate reader (Labtech, UK). The OD values of each seeding number were compared to the other seeding numbers and to the blank control at each of the examined time points (i.e. 24, 48 and 72 h).

### 2.6 Inhibitory effects of *C. sinensis* on trophozoites

We investigated the effect of various concentrations of hot and cold *C. sinensis* brews on the growth kinetics of trophozoites using manual counting and the colorimetric SRB assay. First, 3.2 × 10^5^ trophozoites were seeded in 7 ml PYG medium into T25 flasks that were incubated in a Stuart oven at 25 °C. Then, sterile hot and cold *C. sinensis* brews were added at concentrations of 25%, 50%, 75% and 100%. Negative control included trophozoite culture in PYG only, while the positive control included trophozoite culture in PYG supplemented with 0.02% CHX. After incubation times of 24, 48 and 72 h, trophozoites were counted using a hemocytometer in order to determine the effect of each concentration on the growth rate of the trophozoites. Secondly, the SRB assay was used to examine the *in vitro* growth inhibitory activity of *C. sinensis* against *A. castellanii* trophozoites as described previously (Ortega-Rivas et al., 2016). Briefly, *A. castellanii* trophozoites were seeded at 2.5 × 10^3^ trophozoites/well in 96-well microtiter plates. Each well received 100 μl of the examined concentrations (25%, 50%, 75% or 100%) of hot or cold brew of *C. sinensis*. Negative control wells received only 100 μl PYG medium/well, while positive control wells received 100 μl PYG medium plus 0.02% CHX/well. The plates were incubated in a Stuart oven at 25 °C. Then, at 3, 6, 24, 48 and 72 h post-incubation, the colour absorbance of each well was measured as described above. All experiments were performed in triplicates.

### 2.7 Efficacy of *C. sinensis* brews against cyst formation

Approximately 6.2 × 10^5^ trophozoites were seeded in T25 culture flasks and were treated with 25%, 50%, 75% and 100% *C. sinensis* encystation medium. The negative control included trophozoites treated with standardized encystation medium alone. The positive control included trophozoites treated with standardized encystation medium plus 5% phenyl-methanesulfonyl fluoride (PMSF) solution (Sigma Life Science, Switzerland), which is a serine protease inhibitor. The encystation rate was determined by counting the number of trophozoites *versus* cysts for each concentration after 24, 48 and 72 h of treatment using a hemocytometer. Also, after 72 h, the cultures were treated with 0.5% sodium dodecyl sulphate (SDS) and left for 60 min at ambient temperature to digest any remaining trophozoites. The, pre- and post-SDS digestion counts were performed and the encystment percentage was determined using the formula (% encystment = Post-digestion number/Pre-digestion number × 100). Images were also obtained for each treatment and at each incubation time using a Leica DMIL CMS inverted microscope (Germany) with Leica Application Suite (LAS version 4.3).

### 2.8 Scanning electron microscopy (SEM)

Trophozoites treated with 100% *C. sinensis* were harvested, fixed, and dehydrated using a graded series of ethanol as described below for TEM. After dehydration, critical point drying was performed by infiltrating the samples in hexamethyldisilazane (HMDS) (Acros, Germany) for 5 min twice to further enhance drying of the samples. The samples were mounted on aluminium SEM stubs with a double-sided carbon sticker and sputter coated with gold prior to SEM imaging. Images were obtained using a JEOL JSM 7001F SEM (JEOL, Ltd., Tokyo, Japan).

### 2.9 Transmission electron microscopy (TEM)

Trophozoites treated with 100% *C. sinensis* were fixed in 3% glutaraldehyde in 0.1M Cacodylate buffer for 24 h. After fixation, the samples were washed twice in 0.1M cacodylate wash buffer and re-suspended in 1% osmium tetroxide for 1 h. The samples were washed twice in distilled water and embedded in 3% agarose low gelling for 5 min at 4 °C. This was followed by dehydration using a graded ethanol series of 50, 70, 90 and 100%. The dehydration process was performed as follows; 50% ethanol 10 min twice, 70% for 10 min twice, 90% for 10 min twice, and 100% ethanol for 10 min thrice. Then, 100% propylene oxide was used for 15 min twice as a transitional solvent between ethanol and epoxy resin. After dehydration, the samples were infiltrated with a 3:1 propylene oxide-resin mixture for 1 h and re-infiltrated with a 1:1 propylene oxide-resin mixture for 24 h. Then, samples were re-infiltrated with 100% resin twice for 1 h in fresh bottles before embedding in plastic moulds with 100% resin and placed in an oven at 60 °C for 48 h to polymerize. After polymerization, the samples were sectioned for TEM using an ultra-microtome (Leica EM UC6, Germany), mounted on 3.05 mm Gilder copper grids (TAAB, UK), and stained with Uranyl acetate and lead citrate. Samples were examined and images were obtained by using a JEM2100 TEM (JEOL, Ltd., Tokyo, Japan).

### 2.10 Transient exposure of A. castellanii trophozoites to *C. sinensis* brew

Approximately 3.2 × 10^5^ trophozoites seeded in T25 flasks were treated with 5 ml of graded concentrations (25%, 50%, 75% and 100%) of hot or cold *C. sinensis*-PYG (v/v). After 6 and 24 h post-treatment, trophozoites were harvested, washed twice with PYG medium to remove the traces of *C. sinensis*. Then, trophozoites were suspended in 5 ml of fresh PYG and further incubated for 24, 48 and 72 h. At each of these incubation time points, the number of trophozoites was counted using a hemocytometer in order to determine the growth inhibitory effect of transient exposure of *A. castellanii* trophozoites to each of the examined concentrations. The treated trophozoites were also examined visually using a Leica DMIL CMS inverted microscope (Germany) with a Leica Application Suite (LAS version 4.3) in order to identify any morphological alterations caused by transient exposure to *C. sinensis* brew.

### 2.11 Effect of *C. sinensis* brews on excystation of A. castellanii cysts

Approximately 2 × 10^5^ *A. castellanii* cysts suspended in 2 ml of encystation medium were added into 15 ml falcon tubes. The tubes were centrifuged at 500×*g* for 5 min, the supernatant was discarded, and each resultant pellet was suspended with 5 ml of the following solutions: PYG media (negative control), 0.02% CHX in PYG (positive control) and 25%, 50%, 75% and 100% *C. sinensis* in PYG (v/v) of the cold and hot brews. The suspensions were mixed using a vortex mixer to ensure homogenous mixture. The samples were placed on a rack and incubated in a Stuart oven at 25 °C. At 24, 48 and 72 h post treatment, the number of trophozoites and cysts in each sample was determined using a hemocytometer and a CETI inverted microscope (Medline Scientific, UK). All experiments were repeated three times and the mean values were calculated.

### 2.12 Identification of chemical ingredients in *C. sinensis* brews by UHPLC-MS

Chromatography analysis of hot and cold brews was performed on a Waters Acquity UPLC- Xero G2-XS QTOF Quadrupole Time-of-Flight Mass Spectrometer (Waters, UK). Two solvents (A and B) were used as mobile phase. Solvent A was 0.1% formic acid (FA) in water + methanol at a 90:10 ratio, while solvent B was 0.1% FA in methanol. A Restek C_18_ RaptorTM column (Restek Thames, UK) was used and the injection mode was performed by automatic liquid injection with an injection volume of 1 ml (50 μl of hot or cold *C. sinensis* brew diluted in 950 μl of LC-MS grade methanol). The flow rate was 0.25 ml/min with a run time of 15 min. The gradient elution of solvent B was 10% ramped to 95% over a period of 15 min, while that of solvent A was 95% ramped to 10% over a period of 15 min. The raw data were analyzed and chemical composition of each component was confirmed by matching the chemical formula with data from the library of MassLynx® software (version 4.1, Waters).

### 2.13 Statistical analysis

Data were analyzed using one-way and two-way analysis of variance (ANOVA) as appropriate, while post-hoc direct comparisons were performed using Tukey's multiple comparisons test. All experiments were performed in triplicate and data were expressed as mean ± standard error of mean (SEM). *p* < 0.05 was considered statistically significant. All statistical analysis was performed using GraphPad Prism Version 7.03 (GraphPad Software, Inc. USA).

## 3 Results

### 3.1 Corneal stromal cell toxicity testing

**Fig. 1:**
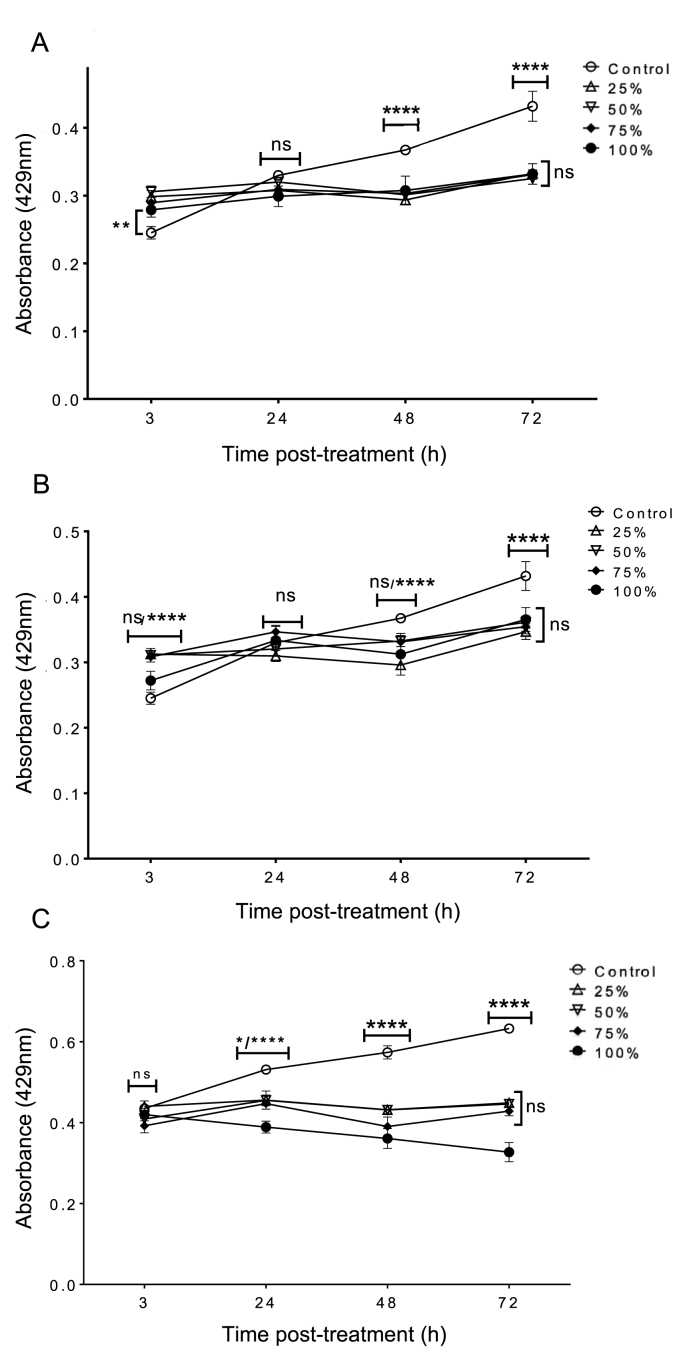
Cytotoxicity of *Camellia sinensis*. Cold (A) and hot (B) brews of *C. sinensis* were separately tested against primary human corneal stromal cells. (C) Cold brew was tested against SV40-immortalized human corneal epithelial cells. * *p* < 0.05; **** *p* < 0.0001, ns, *p* > 0.05.

Primary corneal stromal cells were exposed to serial concentrations of cold and hot *C. sinensis* brews (25%, 50%, 75%, 100%). Dilutions were made using the respective culture medium v/v. The two-way ANOVA of cold brew *C. sinensis* revealed a significant main effect of time (h) (F (3, 60) = 97.01, *p* < 0.0001), concentration (F (4, 40) = 8.084, *p* = 0.0005), and time × concentration interaction (F (12, 60) = 22.77, *p* < 0.0001). Likewise, the two-way ANOVA of hot brew revealed a significant main effect of time (h) (F (3, 60) = 93.98, *p* < 0.0001), concentration (F (4, 20) = 3.079, *p* < 0.05), and time × concentration interaction (F (12, 60) = 13.87, *p* < 0.0001). At 3 h post-treatment, cells treated with all *C. sinensis* concentrations showed a slight increase in cell number compared to untreated control for both brews (Fig. 1A and B). By 24 h post-treatment, there was no significant difference between all *C. sinensis* treated cultures and control cells for both brews. At 48 h post-treatment, the OD values of the control cells increased significantly compared to that obtained at 24 h and were also significantly higher compared to all *C. sinensis*-treated cultures for both brews (*p* < 0.0001). The temporary sluggishness in the growth rate of corneal stromal cells at this time point might be attributed to transient cytotoxicity because all treated cells managed to continue their growth until 72 h. In fact, the growth rate of all treated cells at 72 h was significantly higher than that of the same cells at 48 h. However, the significant difference between the control and treated cells continued to increase in favour of control cells (*p* < 0.0001). The transient replication arrested phase observed at 24 and 48 h did not compromise the ability of the treated cells to resume their growth even at 100% concentrations of both brews. However, this shows that *C. sinensis* had a mild cytotoxic effect on human primary corneal stromal cells.

### 3.2 SV40-immortalized human corneal epithelial cells (iHCECs) cytotoxicity

Here, we determined the toxicity of various concentrations of cold *C. sinensis* brew (25%, 50%, 75%, 100%; v/v) against iHCECs. Two-way ANOVA revealed a significant main effect of time (h) (F _(4, 8)_ = 86, *p* < 0.0001), *C. sinensis* concentration (F _(3, 6)_ = 14.33, *p* = 0.0038), and time × concentration interaction (F _(12, 24)_ = 11.34, *p* < 0.0001). Post-hoc comparisons between the untreated (control) cells and cells treated with the various concentrations of cold *C. sinensis* brew at 3 h showed no significant difference between the control and treated cells. At 24 h post-treatment, the OD values were increased for control cells compared to all treated cells (*p* < 0.05). Also, 100% *C. sinensis* brew caused a significant reduction in the cell growth compared to cells treated with lower concentrations (*p* < 0.05). Between 24 and 72 h, the control cells exhibited progressive increase in the growth rate compared to cells treated with all concentrations (*p* < 0.0001). The 100% *C. sinensis* brew continued to cause gradual decrease in cell growth. Interestingly, the growth rates of cells treated with 25, 50 or 75% *C. sinensis* maintained more or less a similar trend (i.e. no increase or decrease) (Fig. 1C). This result indicates that cold *C. sinensis* brew had a toxic effect against iHCECs, particularly at 100% (i.e. the highest) concentration.

### 3.3 Cytotoxicity of corneal stromal cells using acridine orange (AO) staining

**Fig. 2:**
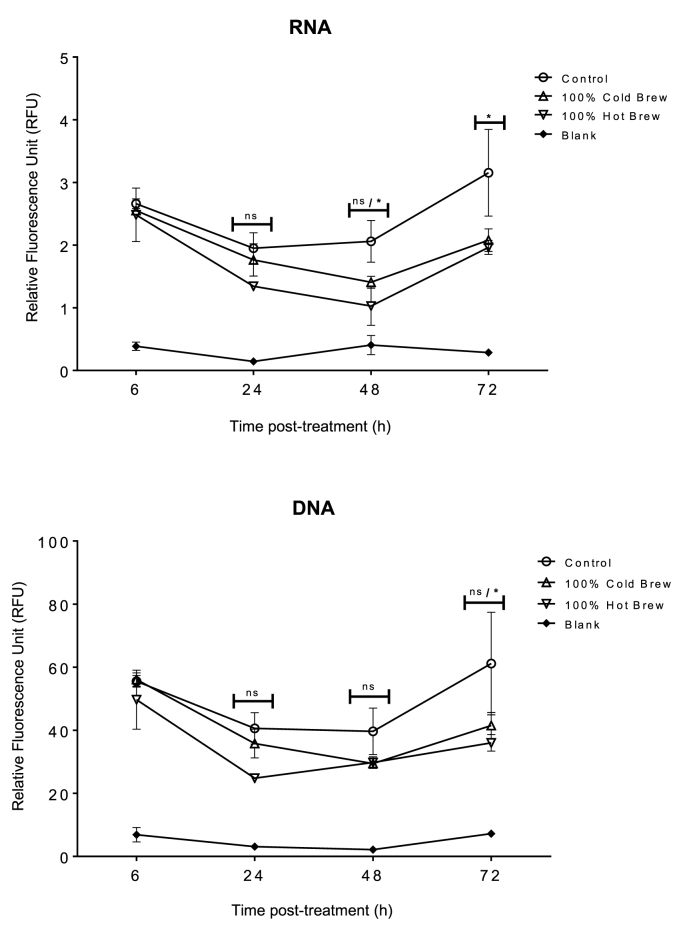
Cytotoxicity of 100% *C.sinensis* brew against primary corneal stromal cells by fluorometric quantification of RNA and DNA contents in corneal cells using acridine orange stain. Although there was a reduction of RNA concentration at 48 h, the trends for both brews showed an increase of RNA concentration at 72 h. There was no significant difference between the DNA concentration of the control and cold brew, however there was a slight difference between the DNA concentration of the hot brew and the control at 72 h post-treatment with *C. sinensis*. * *p* < 0.05; ns, p > 0.05.

For further verification of the lack of sustained cytotoxicity of *C. sinensis* brew forms to corneal stromal cells, fluorometric quantification of RNA and DNA content in the corneal stromal cells post treatment with both, was achieved by using AO straining in a 96-well plate format. When AO binds to DNA, it exhibits an excitation at 502 nm (cyan) and an emission at 525 nm (green), and when it binds with RNA, the excitation is located at 460 nm (blue) and the emission is located at 650 nm. With the excitation and emissions from both nucleic acids, the level of fluorescent emitted can be proportionally related to the number of cells in each treatment sample. As shown in Fig. 2, at 24 h post-treatment of corneal stromal cells with both forms of 100% *C. sinensis*, there was no significant difference between the control (M199 medium-treated culture) compared to both brews, because the concentrations of RNA and DNA were not significantly different (*p* > 0.05). At 48 h, the RNA concentration of the cold brew was not significantly different from the control, while that of the hot brew had a slightly reduced concentration (*p* < 0.05). At 72 h post-treatment, both brews had a lower RNA concentration compared to control. Although there was a reduction of RNA content at 48 h, the trends for both brews showed an increase of RNA content at 72 h. The DNA results show that between 6 h and 72 h, there was no difference between the DNA content of the control and the cold brew, while there was a slight difference between the DNA content of the hot brew and the control at 72 h post-treatment with *C. sinensis*.

### 3.4 Optimal seeding density

Our aim was to examine whether initial seeding density and seeding time can influence trophozoite seeding efficiency, and consequently any subsequent analysis. To determine the optimal seeding density, *A. castellanii* trophozoites at various concentrations (1 × 10^3^, 2.5 × 10^3^, 5 × 10^3^, 7.5 × 10^3^ and 10 × 10^3^ trophozoites/well) were incubated over a period of 72 h. Apart from 1 × 10^3^, all other concentrations grew to confluency by 72 h in 96-well plates. The two-way ANOVA revealed a significant main effect of time (F _(5, 10)_ = 580.9, *p* < 0.0001), trophozoite number (F _(2, 4)_ = 983, *p* < 0.0001), and time x trophozoite number interaction (F _(10, 20)_ = 58.89, *p* < 0.0001). Using the quantitative colorimetric SRB assay for testing *A. castellanii* growth kinetics, we deduced that the optimum seeding density for 96-well plates with the L-T 4000 microplate reader (Labtech, UK) is 2.5 × 10^3^ trophozoites/well. In the course of the experiment, the approximate number of trophozoites for each group was significantly different between each time point of 24, 48 and 72 h, except for 1 × 10^3^ which showed a significant increase in cell numbers only between 48 h and 72 h (Fig. S1).

At 24 h, 10 × 10^3^ showed a significant increase in cell numbers (*p* < 0.0001) in comparison with the other concentrations while 5 × 10^3^ and 7.5 × 10^3^ showed no significant difference in cell number (*p* > 0.05), the same was observed for 1 × 10^3^ and 2.5 × 10^3^ (*p* > 0.05). At 48 h, there was no significant difference in cell number between 7.5 × 10^3^ and 10 × 10^3^ concentrations (*p* > 0.05). However, there was a significant difference between the 7.5 × 10^3^ and 10 × 10^3^ concentrations and the 1 × 10^3^, 2.5 × 10^3^and 5 × 10^3^ concentrations (*p* < 0.0001). At 72 h, there was no significant difference between the 10 × 10^3^ concentration and the 2.5 × 10^3^, 5 × 10^3^ and 7.5 × 10^3^ concentrations, however, the 1 × 10^3^ concentration had a significant difference in concentration in comparison to the higher cell concentrations (*p* < 0.0001). Although individual analysis of 2.5 × 10^3^ and 5 × 10^3^ concentrations showed a significant difference at each time point, the difference between 5 × 10^3^ and 7.5 × 10^3^ at 48 h was slim when compared with the difference between 2.5 × 10^3^ and 7.5 × 10^3^. The trend of replication of 2.5 × 10^3^ in relation to other seeding densities suggests that using 2.5 × 10^3^ as an initial seeding density can clearly delineate the changes in the growth rate of trophozoites in response to treatment compared to the control at each time-point during the course of a 72-h experiment.

### 3.5 Survival of A. castellanii trophozoites to *C. sinensis* hot brew

**Fig. 3:**
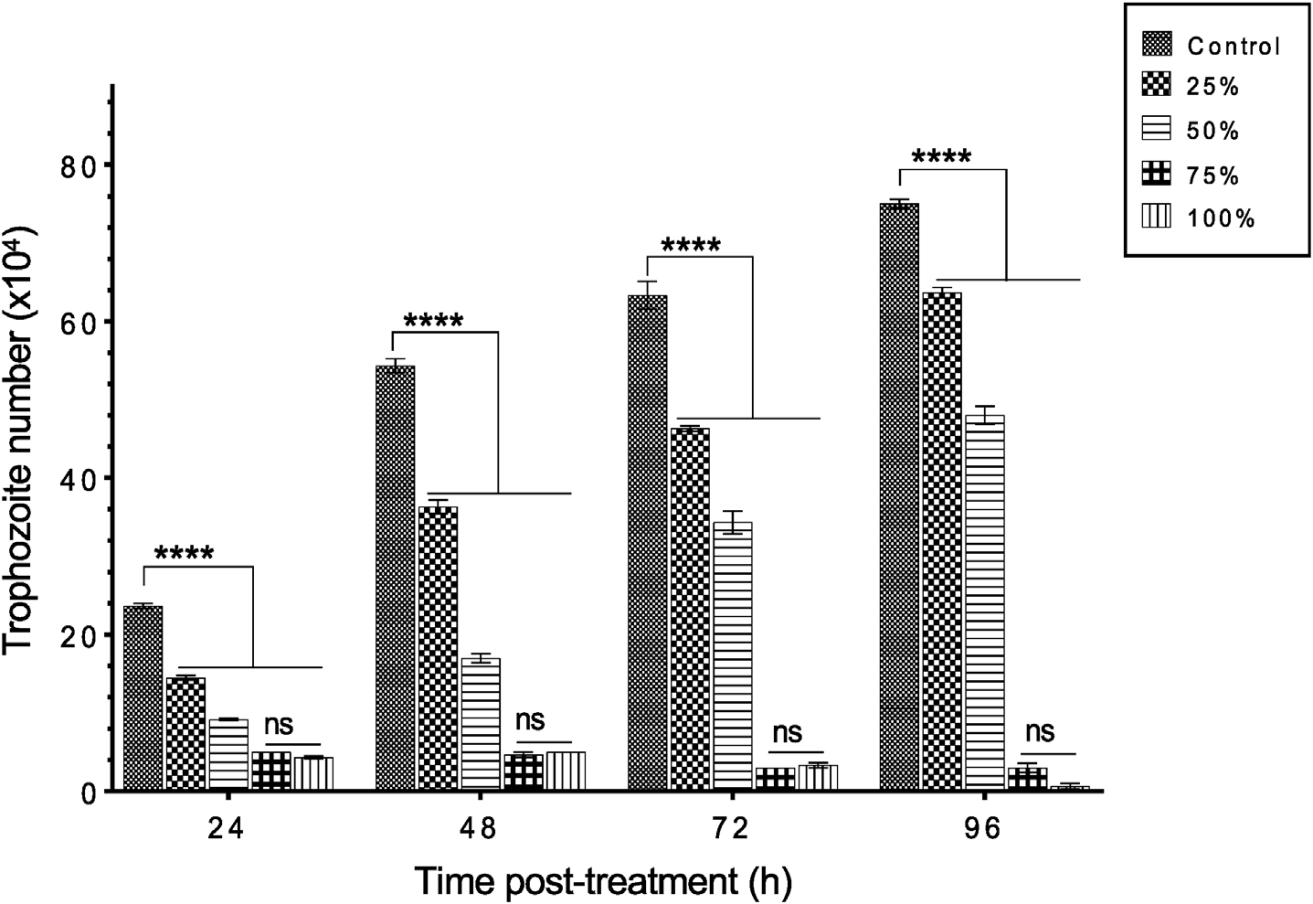
Growth inhibitory effect of *C. sinensis* on *A. castellanii* trophozoites at 24, 48, 72 and 96 h after treatment with 25%, 50%, 75% and 100% of *C. sinensis*. Significant differences were detected at all time points when control was compared to 25%, 50%, 75% and 100% (*p* < 0.0001). The 25% and 50% concentrations showed an increased in trophozoite numbers between 24 and 96 h. There was a progressive decrease in trophozoite number for 75% and 100% concentrations between 24 and 96 h with no significant difference between the effects of 75% and 100% (*p* > 0.05). (****, *p* < 0.0001; ns, *p* > 0.05).

In comparing the effect of *C. sinensis* on the number of trophozoites measured quantitatively by hemocytometer, the two-way ANOVA revealed a significant main effect of time (F (3, 30) = 1610, *p* < 0.0001), treatment (F (4, 10) = 2318, *p* < 0.0001) and a significant time × treatment interaction (F (12, 30) = 361.7, *p* < 0.0001). As shown in Fig. 3, post-hoc comparisons showed a significant increase in trophozoite numbers of the control, and 25% and 50% *C. sinensis* (*p* < 0.0001) between 24 and 96 h post-treatment. At 24 h, the control (untreated) showed a significant increase (*p* < 0.0001) in comparison to all the treated groups. There were some slight differences between 25% and 50% treatment, while there was no significant difference between the effect of 75% and 100% of *C. sinensis* (*p* > 0.05) in the duration of the experiment. The progressive decline of trophozoite number caused by exposure to the two highest concentrations suggests an inhibition of the parasite replication and/or cytolysis. As the experiment progressed, there was an increase in parasite numbers in the control (untreated) and the 25% and 50% (treated groups), whereas the 75% and 100% groups showed approximately the same parasite growth rate between 24 and 48 h time points. The control, 25% and 50% groups showed an increase of trophozoite number by 57, 61 and 44%, respectively.

The direct anti-amoebic effect of hot brew *C. sinensis* between 24 and 72 h on the structure of trophozoites was determined by microscopic examination. At 24 h, cytoplasmic contents of trophozoites were expelled into the culture medium in the form of extracellular vesicles (EVs) (Fig. 2S). More rounded trophozoites were detected in response to 100% hot brew, suggesting the presence of non-adherent trophozoites. At 48 h, more EVs were observed in cultures treated with 25%, 50% and 75% *C. sinensis*. This finding was not observed in culture treated with 100% *C. sinensis* (Fig. 2S). Adhesion to flask surfaces was inhibited by all *C. sinensis* concentrations, which was observed with gentle agitation of the flasks from side to side. At 72 h, significant cytolysis was observed in cultures treated with 25%, 50% and 75% *C. sinensis*, but incomplete and arrested encystation was detected in the culture treated with 100% *C. sinensis*. These results illustrate that not only were trophozoites significantly damaged when treated with 75% and 100% *C. sinensis*, but also the parasite's ability to counter the treatment by differentiating into cysts was inhibited at 100% *C. sinensis*.

### 3.6 Survival of A. castellanii trophozoites in response to hot versus cold brews

**Fig. 4:**
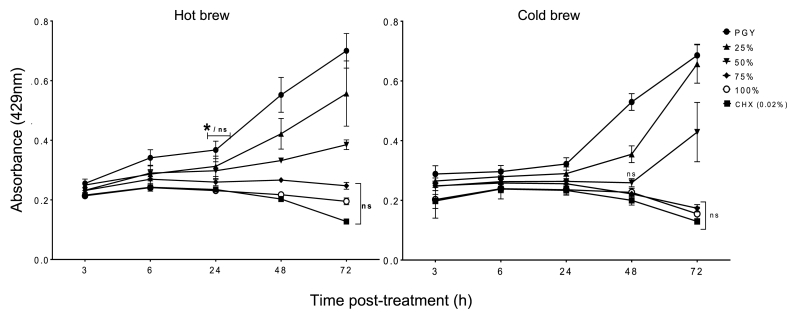
Acanthamoebicidal activity of hot and cold brews of *C. sinensis* against *A. castellanii* trophozoites showed no significant difference between the highest concentrations, 75% (*p >* 0.05), 100% (*p >* 0.05) and chlorhexidine (CHX) at 72 h.

The two-way ANOVA of hot brew *C. sinensis* revealed a significant main effect of time (F _(5, 10)_ = 27.38, *p* < 0.0001), concentration (F _(4, 8)_ = 22.95, *p* < 0.05), and a time × concentration interaction (F _(20, 40)_ = 13.22, *p* < 0.0001). The two-way ANOVA of cold brew revealed a significant main effect of time (F _(5, 15)_ = 648.1, *p* < 0.0001), concentration (F _(4, 12)_ = 208.6, *p* < 0.0001), and a time × concentration interaction between both parameters in the treatment groups (F _(20, 60)_ = 103.9, *p* < 0.0001). Post-hoc comparisons of hot brew effects between 3 and 6 h showed no significant difference between the OD of treatment groups (*p* ≥ 0.05). At 24 h, there was a difference between the control (untreated) (*p* < 0.05) and the treatment groups, while there was no difference between the effect of the treatment groups when compared to each other (*p* ≥ 0.05) (Fig. 4). Between 24 h and 72 h, there was a progressive increase in OD of control (untreated), 25% and 50%. However, there was a significant decrease between the OD of the control and the lower treatment concentrations, suggesting some level of growth inhibition. By contrast, the 75% and 100% concentrations showed a decrease in OD between 24 h and 72 h, without exhibiting any significant difference between their effect and that of CHX (*p* > 0.05).

For the cold brew experiment, the trend observed was similar to the effect of the hot brew. Between 3 and 24 h, there was a slight increase in the OD of the control (untreated) compared to treatment concentrations, while the treatment concentrations maintained the same effect for 24 h. At 48 h, there was a significant difference between the control (untreated) (*p* < 0.05) and treatment groups, and also a significant increase of OD between the 25% concentrations and the 50, 75 and 100% concentrations. The 50, 75 and 100% concentrations showed no significant difference between their activities (OD) (*p* > 0.05) and also no significant difference was observed between their activity and that of CHX (*p* > 0.05) (Fig. 4). This trend continued for the 75% and 100% concentrations, while the 50% concentration showed a significant increase between its OD and that of the higher concentrations (*p* < 0.05).

As observed in the results of both hot and cold *C. sinensis* treatment, all groups showed either a lag phase or slight replication between 6 h and 24 h. At 25% a similar activity trend as the control was detected and with continuous replication till the end of experiment. 100% and 75% groups inversely showed continuous acanthamoebicidal activity between 6 h and 72 h with no significant difference between 100% and CHX (*p* < 0.05). These results show that over a period of 72 h post-exposure of *A. castellanii* to serial concentrations of hot and cold *C. sinensis*, there was a concentration-dependent acanthamoebicidal activity and inhibition of the parasite's replication.

### 3.7 Ultrastructural characteristics of C. sinensis-treated trophozoites

**Fig. 5:**
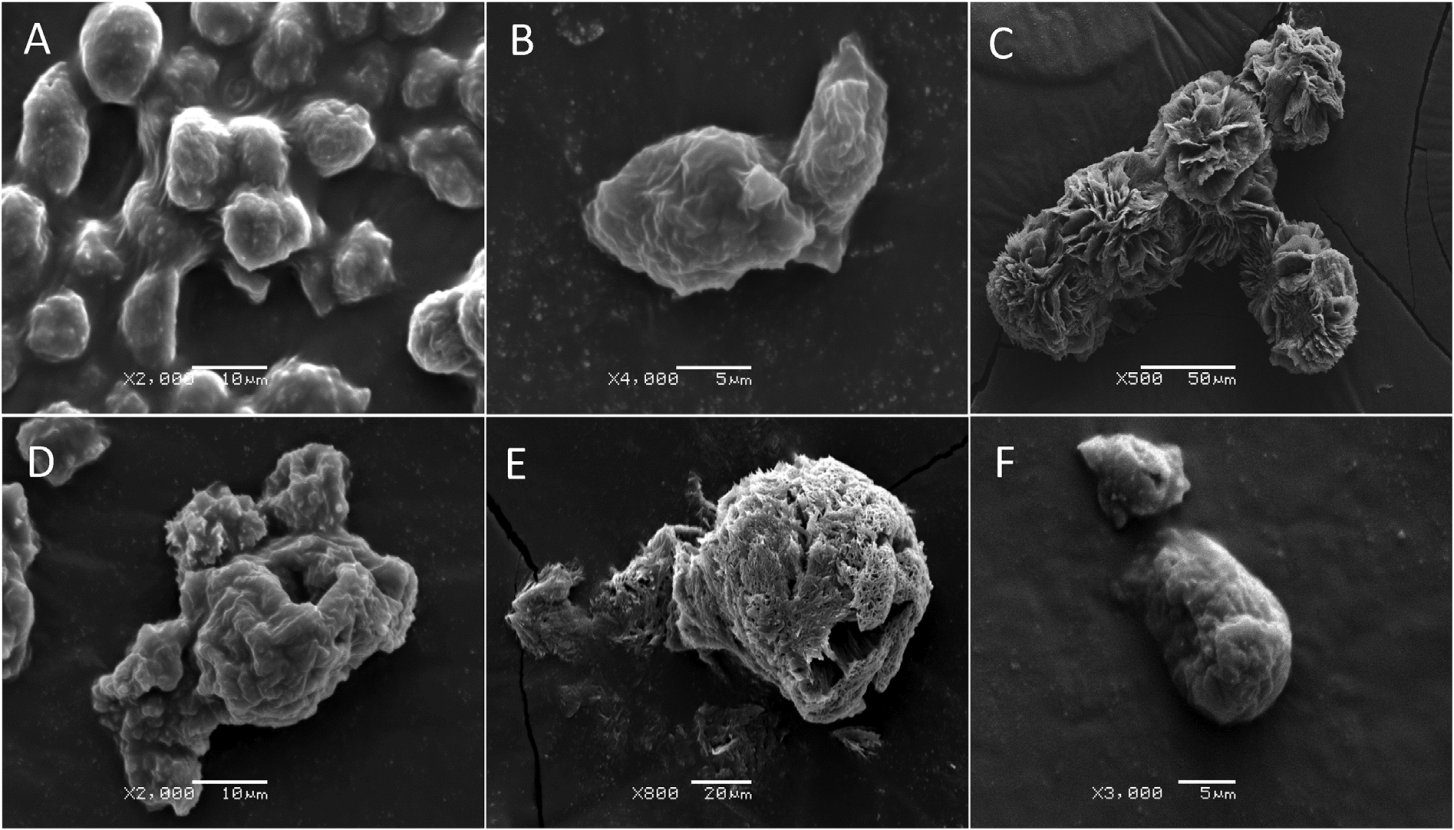
Scanning electron micrographs of *A. castellanii* treated with *C. sinensis.* Continued exposure to both chlorhexidine (CHX) and hot *C. sinensis* showed loss of acanthopodia and progressive destruction of *A. castellanii* trophozoites. (A) A group of trophozoites cultured in PYG medium (control) attached together and adherent to the surface; (B) Closer view of two trophozoites in PYG (C) Trophozoites in CHX for 24 h. (D) Trophozoites in hot *C. sinensis* for 24 h are misshaped with cell membrane disruption; (E) Membrane perforations and loss of integrity in trophozoites treated with CHX for 72 h; (F) Trophozoites in hot *C. sinensis* brew for 72 h are misshaped and shrunken. With increased duration of exposure to *C. sinensis* trophozoites became less adherent to the surface and had reduced cell volume possibly in the attempt to encyst. Magnifications: X2,000, X4,000, X500, X2,000, X800 and X3,000 for A to F, respectively. Scale bars = 10, 5, 50,10, 20 and 5 μm for A to F, respectively.

**Fig. 6:**
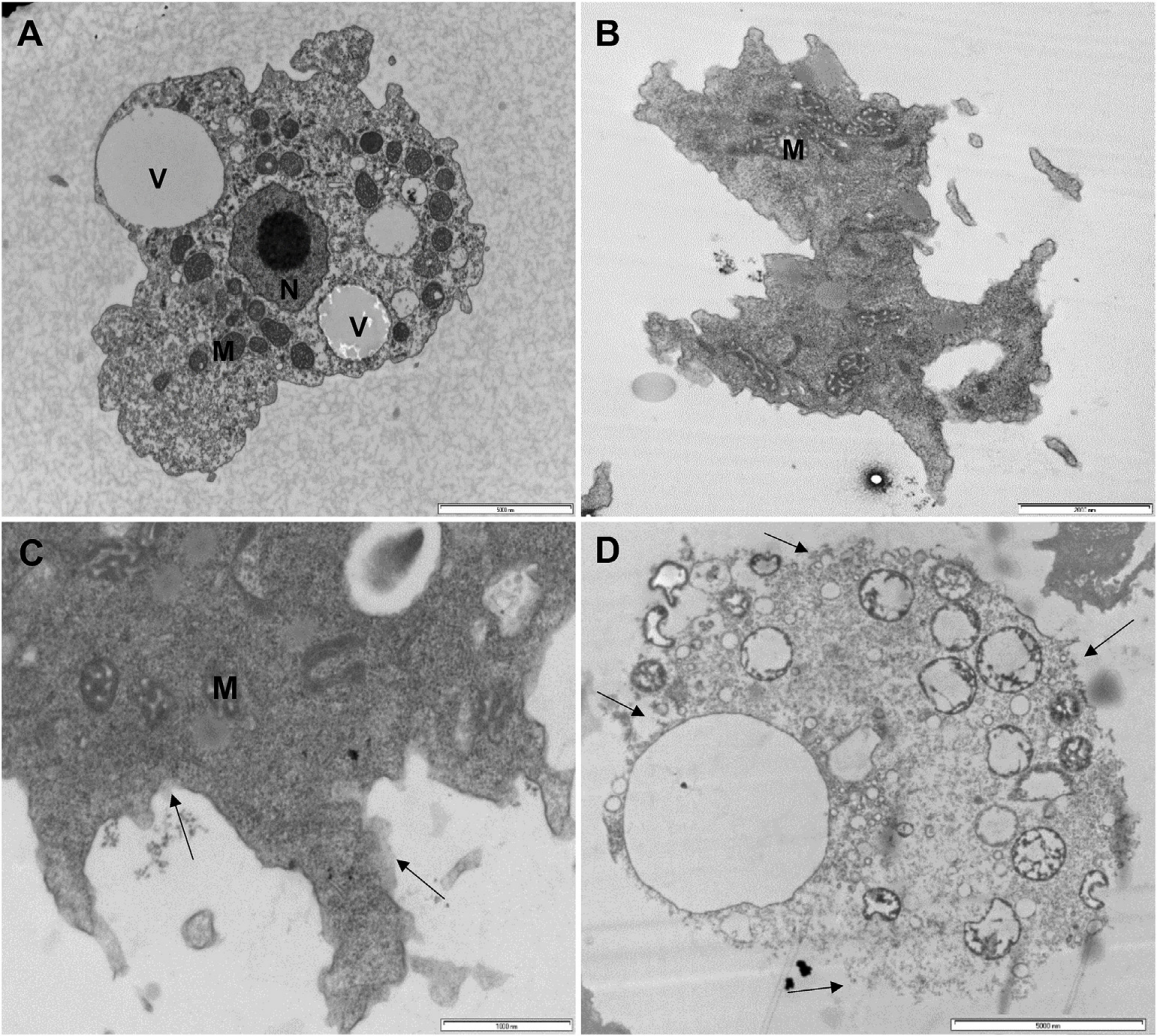
Transmission electron microscopy observations show progressive destruction of *A. castellanii* trophozoites exposed to hot *C. sinensis*. (A) Trophozoite in control PYG medium; (B) Trophozoites in 100% *C. sinensis* at 24 h showing loss of cellular membrane and the acanthopodia; (C) Trophozoites at 48 h exhibit some damage of membrane integrity (arrows); (D) At 72 h post-exposure to *C. sinensis*, trophozoites lost cellular membrane integrity. *Abbreviations:* (V) food vacuole, (M) mitochondria, (N) Nucleus. Magnifications: X6,000, X8,200, X16,500 & X4,200 for A to D, respectively. Scale bars = = (A) 5000 nm; (B) 2000 nm;; (C) 1000; (D) 5000 nm.

SEM revealed progressive morphological alterations and destruction of *A. castellanii* trophozoites due to treatment with *C. sinensis* (Fig. 5). At 24 h post treatment, trophozoites had lost their ability to adhere to each other and started to shrink. With increased exposure, the trophozoites develop abnormal shape and finally become fragmented. Comparison with the results of trophozoites exposed to CHX showed the same morphological changes. Ultrastructural changes within the trophozites were examined using TEM and also confirmed the progressive destruction of *A. castellanii* trophozoites treated with *C. sinensis* (Fig. 6). At 24 h, loss of extracellular material was observed, which progressed to loss of cellular membrane integrity at 48 h and by 72 h destruction of the cellular membrane and cytoplasmic damage were evident.

### 3.8 Transient effect of *C. sinensis* brew against A. castellanii trophozoites

Post-hoc comparisons for 6 h exposure showed no difference (*p* > 0.05) between the parasite growth inhibition caused by CHX and 100% *C. sinensis* cold brew (43% and 36%, respectively) at 24 h, with a significant increase (*p* < 0.0001) of growth inhibition between CHX and the hot brew. The two-way ANOVA of 6 h transient exposure of *A. castellanii* to *C. sinensis* revealed a significant main effect of time (h) (F (2, 6) = 64.65, *p* < 0.0001), concentration (F (2, 3) = 677, *p* < 0.00001), and time × concentration interaction (F (4, 6) = 32.11, *p* = 0.0003). The two-way ANOVA of 24 h transient exposure revealed a significant main effect of time (h) (F (2, 6) = 233.4, *p* < 0.0001), concentration (F (2, 3) = 786.6, *p* < 0.00001), and a time × concentration interaction (F (4, 6) = 113.5, *p* < 0.0001). At 48 h, there was an increase of growth inhibition of 93% for the CHX with significant increase (*p* < 0.0001) to cold and hot brews that had 44% and 41% growth inhibition, respectively. The trophozoite growth inhibition of CHX at 72 h was 97%, suggesting significant increase between growth inhibition caused by CHX compared to that of cold and hot brew *C. sinensis* (*p* < 0.0001), that caused 19% and 18% growth inhibition, respectively.

**Fig. 7:**
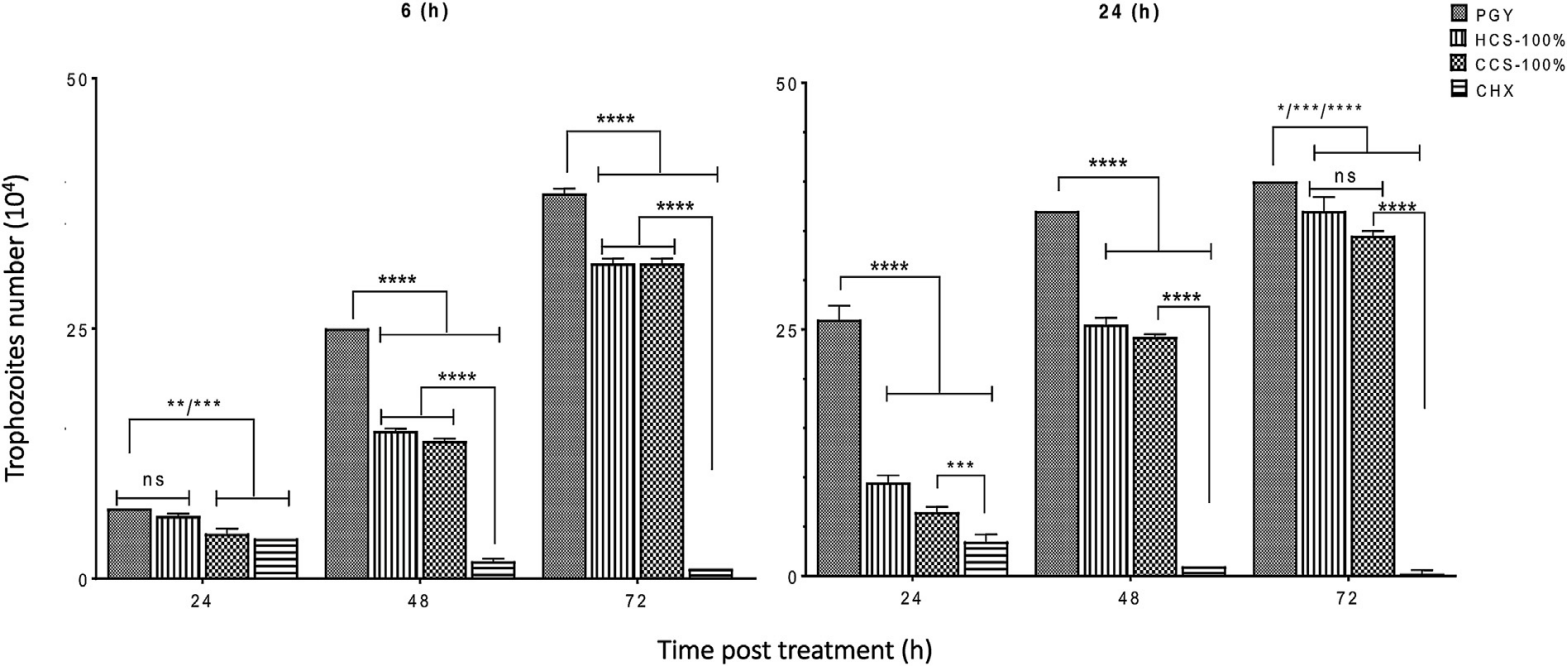
Growth inhibition of trophozoites post-transient exposure to 100% *C. sinensis* brews (cold [CCS] or hot [HCS]) and chlorhexidine (CHX) for 6 and 24 h. Significant increase in inhibition of the trophozoite growth was caused by exposure to CHX compared to cold and hot brews (*p* < 0.0001). (** *p* < 0.05, **** *p* < 0.0001, ns, *p* > 0.05).

Post-hoc comparisons for 24 h exposure showed parasite growth inhibition of 74.8%, 63.3%, and 86.4% for cold and hot brew *C. sinensis* and CHX, respectively. There was a significant increase in growth inhibition between the cold to hot brew and CHX (*p* < 0.05), and hot brew to CHX (*p* < 0.0001). The parasite growth inhibition for CHX increased at 48 h and subsequently at 72 h by 97.3% and 99.3%, respectively, while there was a sharp decline for the cold and hot brew *C. sinensis* at 48 and 72 h from 34.4% to 31% and 13.7% to 7.5%, respectively. The growth inhibitory effect of cold and hot showed had no significant difference (*p* > 0.05) with a significant decrease of inhibition (*p* < 0.0001) of both effects compared to that of CHX. These results (Fig. 7) indicate that over a period of 72 h post-transient exposure of *A. castellanii* for 6 and 24 h, respectively, there was an increased inhibition of parasite growth for cold and hot *C. sinensis* between 24 and 48 h, which declined between 48 and 72 h, while CHX displayed progressive inhibition of parasite growth for 6 and 24 h exposure.

### 3.9 Inhibition of encystation

**Fig. 8:**
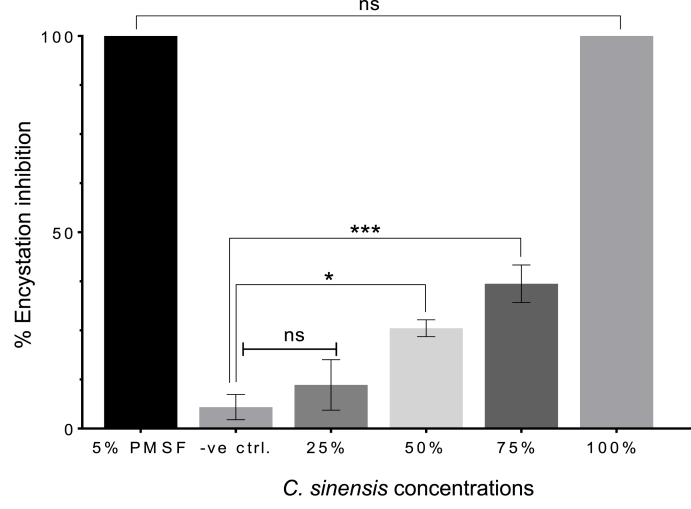
Percentage inhibition of *A. castellanii* encystation. At the highest concentration, 100% *C. sinensis* encystation medium, no encystation was detected post SDS digestion. The 100% *C. sinensis* medium exhibited significant inhibitory effect on encystation, with no significant difference compared to the positive control (5 mM PMSF) (*P >* 0.9999). (* *p* = 0.0195, *** *p =* 0.0006, ns, *p* > 0.05).

When comparing the effect of *C. sinensis* on inhibition of *A. castellanii* encystation, one-way ANOVA revealed a significant main effect of *C. sinensis* concentration (F (5, 12) = 139.6, *p* < 0.0001) on the inhibition rate. Post-hoc comparisons showed a significant increase in encystation inhibition between positive control (5 mM PMSF) and negative control, 25%, 50% and 75% *C. sinensis* groups (*p* < 0.0001). There was no significant difference between the positive control and 100% *C. sinensis* (*p* > 0.9999). The 100% *C. sinensis* concentration exhibited the same activity as the positive control. The positive control and the 100% concentration caused 100% inhibition of *A. castellanii* encystation. The control (untreated) and 25% concentration did not inhibit encystation (0%), while the 50% and 75% concentrations exhibited 28.6% and 42% inhibition of encystations, respectively (Fig. 8). These results indicate that apart from the lowest concentration (25%), all the treatment concentrations caused inhibition of *A. castellanii* encystation.

### 3.10 Microscopic analysis of *C. sinensis* effect on encystation

Microscopic observations showed that within 1 h into encystation, the control sample showed normal encystation, but samples treated with 25%, 50%, 75% and 100% concentrations of hot *C. sinensis* brew and positive control showed signs of cellular damage. At 2 h, the positive control (PMSF-treated) culture, and those treated with 50%, 75% and 100% concentrations showed increased levels of damage and by 4 h, all treatment groups except the negative control showed fragments in the culture media. At 12 h (Fig. S3), aggregates were observed in the control sample, while the PMSF- and *C. sinensis*-treated cultures showed signs of cellular degeneration characterised by the presence of particles in the encystation media. At 24 h, the control (untreated) showed normal encystation with well-defined cysts, whereas the PMSF-treated culture and those treated with 25%, 50%, 75% and 100% *C. sinensis* showed more damage. There were different sizes of cysts with some approximately as large as 50 μm. This trend progressed through 48 h and by the end of 72 h (Fig. S3) there was clear cellular damage.

*C. sinensis* concentrations initially triggered encystation within the first few hours, which was sustained at subsequent time points of 12, 24, and 72 h (Fig. S3), but subsequently cell death occurred which was more evident in sample treated with 100% *C. sinensis*. At 72 h post induction of encystation, total destruction of the amoeba was evident in the positive control group. This damage was also detected in the 100% *C. sinensis* concentration with cellular fragments bounded together. While cell destruction progressed from 48 h in 25%, 50% and 75% concentrations, cyst aggregates were observed in the negative control, 25% and 50% concentrations at varying degrees. Quantification of the approximate number of cysts per aggregate as displayed (Fig. S3) showed that there was a progressive increase of cysts in the control (standardized encystation medium) whereas, there was a progressive decrease of cysts in the *C. sinensis*-treated encystation samples (Table 1).

**Table 1:** The number of *A. castellanii* cysts per aggregate during encystation in the presence of serial concentrations of *C. sinensis*.

Time (h)	Positive control^a^	Negative control^b^	*C. sinensis* concentrations^b^			
			25%	50%	75%	100%
24	0	4-100 (52 ± 28.15)	2-50 (26.02 ± 14.26)	2-15 (8.5 ± 4.18)	2-10 (6 ± 2.74)	2-10 (6 ± 2.74)
48	0	4-500 (252 ± 143.62)	2-30 (16 ± 8.51)	2-10 (6 ± 2.74)	2-5 (3.5 ± 1.29)	2 (2 ± 0)
72	0	4-500 (252 ± 143.62)	2-30 (16 ± 8.51)	2-8 (5 ± 2.16)	2-3 (2.5 ± 0.71)	0

a Treated with 5% PMSF.

b Results are presented as average (mean ± SD).

### 3.11 Ultrastructural changes of encysting amoeba exposed to *C. sinensis*

**Fig. 9:**
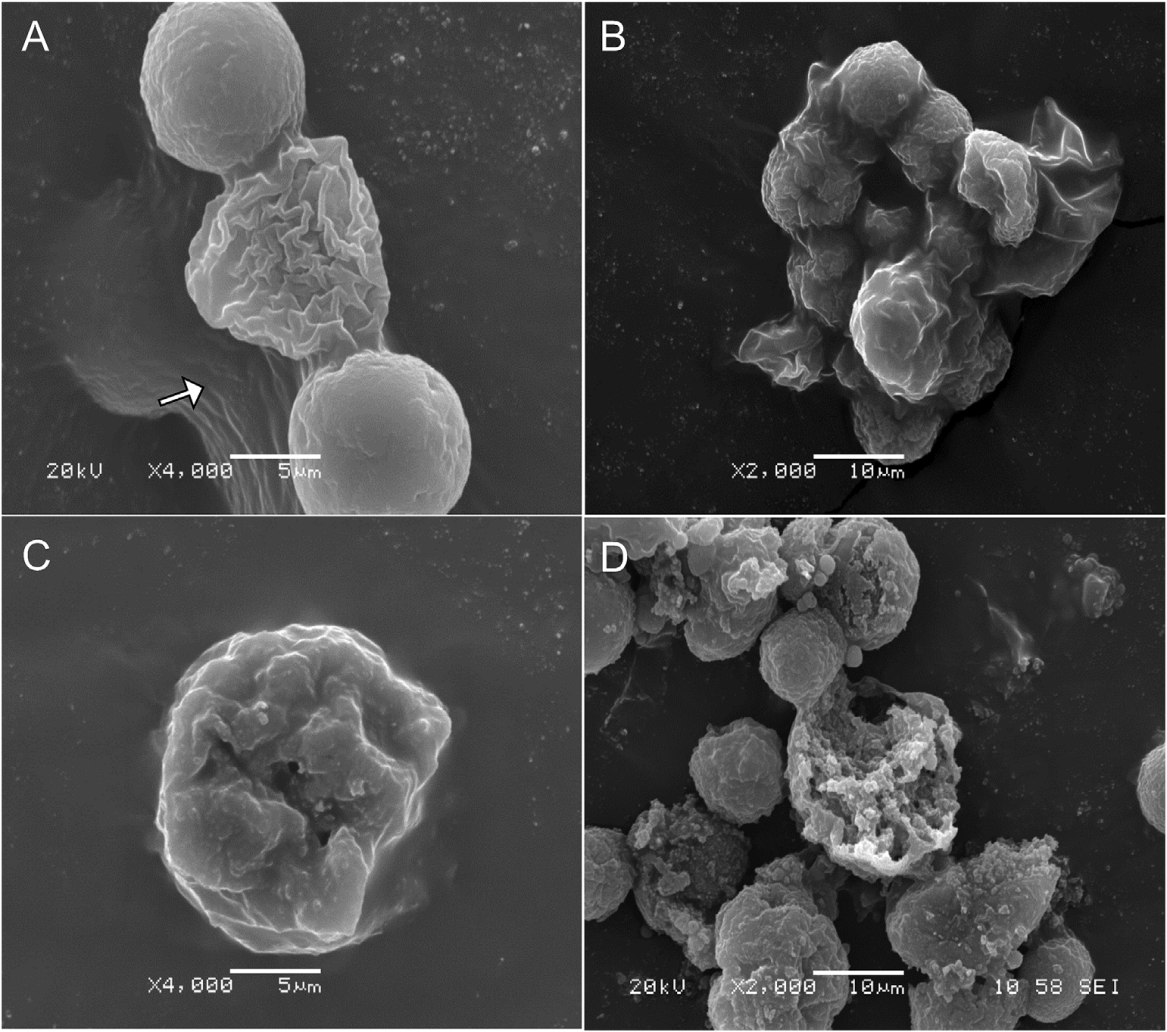
Scanning electron micrographs of *A. castellanii* exposed to *C. sinensis* during encystation. (A) Cysts in the control encystation medium attached together likely by adhesin (arrow); (B) Aggregated cysts exposed to *C. sinensis* at 3 h; (C) Cyst at 24 h shows loss of cellular integrity and membrane perforation; (D) Progressive destruction of cysts at 72 h post-exposure to *C. sinensis*. Magnifications: X4,000 (A and C) and X2,000 (B and D). Scale bars = 5 μm (A, C) and 10 μm (B, D).

**Fig. 10:**
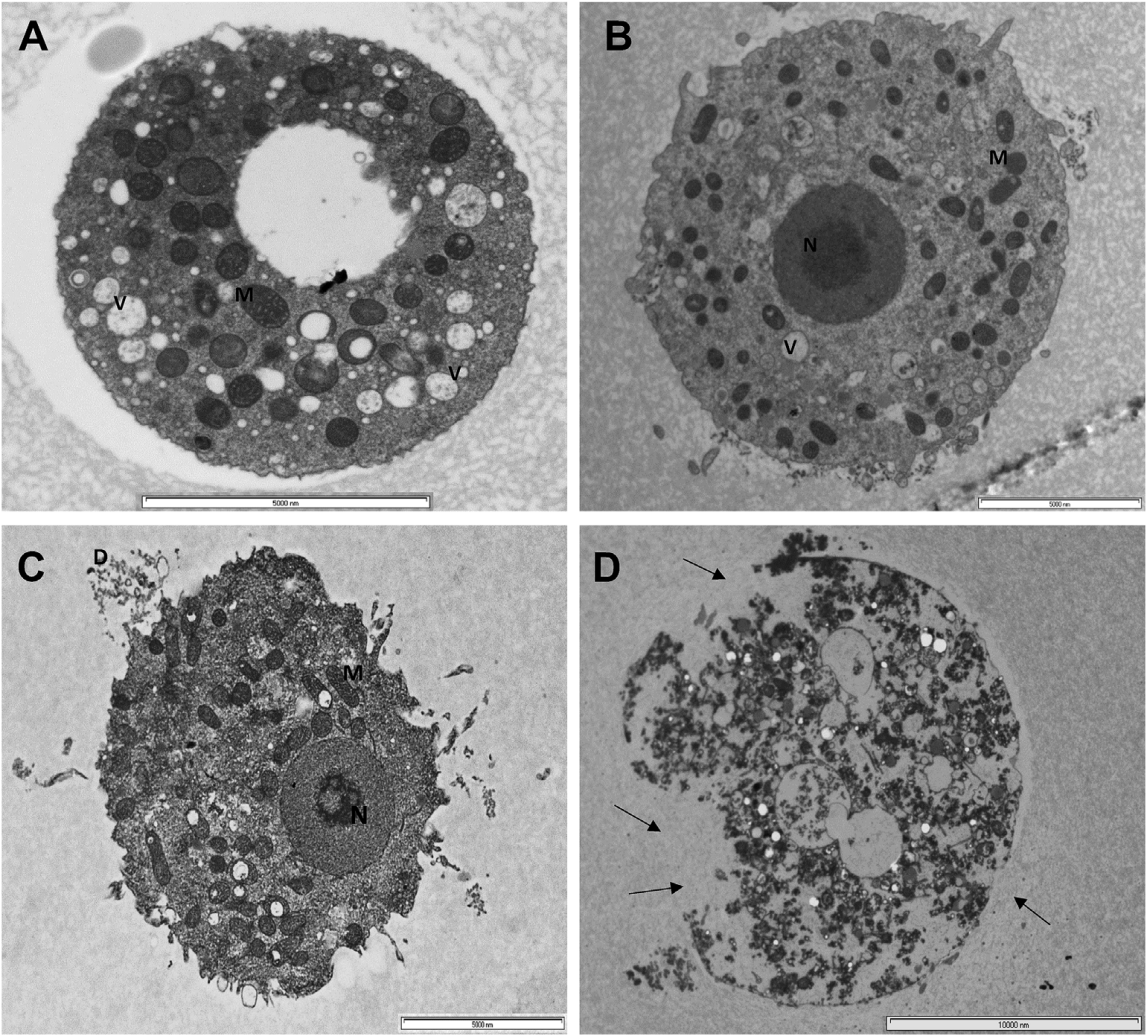
Transmission electron micrographs of *A. castellanii* exposed to *C. sinensis* during encystation. (A) Cyst developed in a control encystation medium where distilled water was used as a solvent. (B) Cyst exposed to 100% *C. sinensis* encystation medium in which cold *C. sinensis* brew was used as the solvent. After 3 h exposure in 100% *C. sinensis* encystation medium, *A. castellanii* cyst had fewer vacuoles (V) and condensed mitochondria (M). (C) At 24 h ultrastructural changes became more discernible with a higher electrodensity in the cytoplasm and less chromatin in the nucleus (N). (D) Pronounced cellular destruction and loss of cellular contents and membrane integrity (arrow) were observed at 72 h post-exposure. The cell volume of the amoeba seems to decrease in a time-proportional manner. Magnifications: X6,000, X4,200, X4,200, and X2,550 for A to D, respectively. Scale bars = 5000 nm (A, B, C) and 10,000 nm (D).

SEM micrographs showed that cysts that were initially aggregated began to lose their adhesive ability over the course of exposure to *C. sinensis* during encystation. As observed in Fig. 9, *C. sinensis* caused a progressive destruction of cysts, while in the control culture, cysts remained in aggregates. At 72 h post-exposure to *C. sinensis*, there was significant destruction of cysts. TEM analysis of the internal ultrastructural alterations in the encysting amoeba showed time-dependent progressive destruction of the cysts (Fig. 10). Compared to *A. castellanii* encysting in normal encystation medium (Fig. 10A), *A. castellanii* exposed to encystation medium prepared in 100% *C. sinensis* brew as the solvent showed less intact membrane, fewer cytoplasmic vacuoles and condensation of the mitochondria at 3 h post-exposure (Fig. 10B). These changes became more obvious in addition to abnormally condensed chromatin by 24 h (Fig. 10C). At 72 h post-exposure, pronounced cellular destruction and loss of membrane integrity were detected together with the presence of fragments of the damaged cysts in the encystation medium (Fig. 10D). Also, with increased duration of exposure to *C. sinensis*, a reduction in the volume of the cysts was observed. These ultrastructural alterations of the encysting trophozoites suggest that the presence of *C. sinensis* in the encystation medium induced some sort of stress and apoptosis-like death of the amoeba and disrupted the organism's ability to form cysts.

### 3.12 Effect of *C. sinensis* on excystment of A. castellanii cysts

In the presence of low concentrations of hot or cold *C. sinensis* brews, more *A. castellanii* cysts were able to differentiate into trophozoites. However, at higher concentrations there were fewer trophozoites compared to the number of cysts that remained intact (Fig. 4S).

### 3.13 LC-MS profile of hot and cold *C. sinensis* brews

UHPLC–QTOF–MS was used to identify the chemical constituents present in hot and cold *C. sinensis* brews. Our chromatographic analysis showed that both *C. sinensis* brew forms contain the same number and type of chemical compounds, in slightly different concentrations (Table 2).

**Table 2:** Chemical constituents of hot and cold *C. sinensis* brews analyzed by UHPLC–QTOF–MS.

Name of the Compounds	Molecular Formula	Molecular Weight (g/mol)	Chemical Class
Caffeine	C_8_H_10_N_4_O_2_	194.19	Methylxanthine alkaloid
Epicatechin gallate	C_22_H_18_O_10_	442.37	Flavanoid
Epigallocatechin gallate	C_22_H_18_O_11_	458.372	Polyphenol
Theogalline	C_14_H_16_O_10_	344.27	Polyphenol
Quercetin	C_15_H_10_O_7_	302.236	Flavanoid
Kaempferol	C_15_H_10_O_6_	286.23	Flavanoid

## 4 Discussion

Considering the need for viable medications against *A. castellanii* infection, and the extensive biological functions and pharmacological activities of green tea, we were motivated to investigate the anti-acanthamoebic activity of *C. sinensis* hot and cold brews against the trophozoite and the cystic forms of this parasite. First, cytotoxicity assays using corneal cells were conducted, bearing in mind that the cornea is the predilection site for AK (Martinez and Visvesvara, 1997; Chomicz et al., 2010), for which CHX digluconate (0.02%) or PHMB (0.02%) drugs are often used. These reference drugs are known to exhibit toxicity when used individually at the regular concentrations, but when used synergistically *in vitro,* their toxic effect was not observed (Mafra et al., 2013). Interestingly, in our study, neither cold nor hot brew of *C. sinensis* at any tested concentrations had a persistent cytotoxic effect against primary corneal stromal cells according to the results obtained by SRB assay. Although high concentrations *C. sinensis* might cause initial inhibition of the growth rate of corneal stromal cells, this effect was not sustained as cells continued to grow to the end of the 72-h assay period. However, the cytotoxic effect of cold brew of *C. sinensis* was more pronounced in SV40-immortalized human corneal epithelial cells, especially at 100% concentration.

Corneal stromal cells are primary cells, whereas corneal epithelial cells are derived from an immortalized cell line. Corneal epithelial cells divide at a higher rate than the primary corneal stromal cells. The differential sensitivity of both cell types to treatment with *C. sinensis* might be attributed to the cell growth rate and culture conditions, or to cell type-specific innate properties that may have contributed to the observed corneal epithelial cell sensitivity. It is also possible that the results observed in long-term assays for the corneal epithelial cells might be influenced by altered sensitivity during progression through the cell cycle; however, this requires further investigation. The cytotoxicity results obtained by Acridine Orange staining assay showed that despite the reduced concentrations of RNA and DNA in 100% *C. sinensis*-treated corneal stromal cells, there was no sustained persistence of cytotoxicity up to 72 h of treatment.

*A. castellanii* has a rapid growth rate especially under *in vitro* conditions (Byers et al., 1991; Siddiqui and Khan, 2012). Therefore, it was necessary to optimize the initial seeding density with respect to the assay design and equipment used under the experimental conditions implemented in the present study. We monitored the temporal changes in the proliferation of *A. castellanii* trophozoites with different initial seeding densities under normal culture conditions (i.e. without any treatment) for up to 72 h. A previous study (Ortega-Rivas et al., 2016) used the SRB assay on a BioTek's PowerWave XS absorbance microplate reader at 562 nm and 630 nm wavelengths, and determined 500 trophozoites/well of the 96-well plate as an optimal seeding density. Using the same SRB assay and L-T 4000 microplate reader (Labtech, UK) at 492 and 630 nm wavelengths wavelength, 2.5 × 10^3^ trophozoites/well of the 96-well plate provided the optimal seeding density under the experimental conditions in our study.

With respect to the anti-acanthamoebic activity of *C. sinensis* against the trophozoites and cysts, our results demonstrated that *C. sinensis* brews are potent inhibitors of *A. castellanii* trophozoite replication *in vitro*. The level of acanthamoebicidal activity exhibited by 75% and 100% *C. sinensis* was comparable to that produced by CHX treatment (Fig. 4). Our data also suggest that the inhibitory effect of treatment with *C. sinensis* brew on trophozoite proliferation was dependent on the concentration of the brew and duration of treatment. Transient exposure to *C. sinensis* for 6 or 24 h was not sufficient to cause irreversible growth impairment of trophozoites (Fig. 7). The cystic stage of *A. castellanii* presents a great challenge to anti-acanthamoebic therapy due to its innate resistance to most drugs. In this study, *C. sinensis* brews have shown a significant cysticidal activity by inhibiting the ability of *A. castellanii* trophozoites to differentiate into mature cysts (Fig. 8). Additionally, *C. sinensis* brews significantly reduced the excystment rate of the amoeba from cysts to trophozoites (Fig. S4).

*C. sinensis* seems to have more apparent toxicity toward human corneal cells compared to *A. castellanii*. This result is anticipated because *A. castellanii* is a resilient organism and has a remarkable ability to adjust to changing environmental conditions and adapt to various stressors (Clarke et al., 2013). Additionally, *C. sinensis*-PYG medium used in anti-*A. castellanii* assays was prepared by dissolving the ingredients of PYG medium in *C. sinensis* brew. This has ensured that *A. castellanii* trophozoites have received all the nutritional needs that they normally obtain from PYG medium and thus can maintain homeostasis. By contrast, cytotoxicity testing against human cells involved the use of *C. sinensis* brew at 100% concentration or diluted with cell culture medium (M199 medium for CSCs and Epilife growth medium for iHCECs) to prepare lower concentrations. Maintenance of mammalian cells *in vitro* depends on the presence of serum, growth factors and many other nutrients in the cell culture medium, which are essential for maintaining the physiology, metabolism and viability of cultured cells (Ishii et al., 2004; Kryczka et al., 2012). Therefore, inhibition of the growth of corneal cells and their apparent sensitivity to *C. sinensis* might have been confounded or aggravated by the scarcity of nutrients in the culture medium used in cytotoxicity testing.

The EVs are known to be produced and secreted by *A. castellanii* (Gonçalves et al., 2018). In this study, the presence of EVs in cultures treated with 25%, 50% and 75% *C. sinensis*, and their absence from cultures treated with 100% *C. sinensis* (Fig. 2S) deserves further reflection. *A. castellanii* secretes several proteins involved in oxidative metabolism and cellular stress, which may play roles in the organism's adaptation to nutritional stress (Gonçalves et al., 2018). Also, *A. castellanii* secretes many proteases, which play roles in its virulence and pathogenesis (Khan et al., 2000), and in the differentiation of the amoeba from trophozoites to cysts (Alsam et al., 2005; Dudley et al., 2008; Lorenzo-Morales et al., 2015). Therefore, the expulsion of cytoplasmic EVs might be a defense mechanism used by *A. castellanii* trophozoites to alleviate the stress caused by *C. sinensis*. On the other hand, exposure of trophozites to 100% *C. sinensis* may have overwhelmed their capacity to adjust to the stress related to *C. sinensis* treatment by expelling these vesicles. Alternatively, the amoeba seemed to use another mechanism to defend its internal contents against such acute stress by rounding up soon after *C. sinensis* treatment as if they were trying to encyst to counter the toxic effect of high concentration of *C. sinensis*. This observation is consistent with previous studies that have shown that *A. castellanii* trophozoites rapidly differentiate into pseudocysts upon exposure to organic solvents (Kliescikova et al., 2011) or contact lens solutions (Kliescikova et al., 2011b), which enable *A. castellanii* to survive such lethal conditions. *A. castellanii* has many proteins that are involved in the modulation of the cellular response to external stimuli (Clarke et al., 2013), providing more evidence to support the ability of this parasite to modify its response and adapt to cellular stress according to the external cues.

Previous studies have presented data on the anti-microbial effects of *C. sinensis* and its components against Gram positive and Gram negative multi-drug resistant pathogens (Parvez et al., 2019). In regard to parasites, *C. sinensis* potentiated the antimalarial effect of artemisinin without interfering with the folate pathway (Sannella et al., 2007; Thipubon et al., 2015). Also, *C. sinensis* reduced the *Haemonchus contortus* worm burden (Zhong et al., 2014), inhibited promastigote and amastigote forms of *Leishmania braziliensis* (Inacio et al., 2014), and inhibited *Leishmania amazonensis* (dos Reis et al., 2013; Inacio et al., 2013). Additionally, *C. sinensis* had inhibitory activity against *Babesia* spp. (Aboulaila et al., 2010), *Eimeria* spp. (Jang et al., 2007) and *Trypanosoma cruzi* (Paveto et al., 2004). Given the broad-spectrum antimicrobial activity of *C. sinensis*, it is important to understand the mechanism by which *C. sinensis* exerts its antiinfective effects because this may lead to more opportunities for the development of better nutraceutical formulations.

How *C. sinensis* exerts its inhibitory effects against *A. castellanii* remains to be elucidated. However, our chromatographic analysis identified caffeine, epicatechin gallate, epigallocatechin gallate, theogalline, quercetin, and kaempferol in the brewed tea. Some of these compounds might have a role in the amoebicidal activity of *C. sinensis*. For example, EGCG has shown antifolate activity against *Stenotrophomonas maltophilia* (Navarro-Martínez et al., 2005), via inhibiting dihydrofolate reductase (DHFR), a key enzyme that catalyzes the reduction of 7,8-dihydrofolate to 5,6,7,8-tetrahydrofolate, which is involved in nucleotide biosynthesis. Therefore, inhibition of DHFR can cause disruption of DNA synthesis. *A. castellanii* has DHFR and the antifolate trimethoprim drug has been shown to have amoebicidal activity (Siddiqui et al., 2016). Therefore, it is possible that EGCG, via its antifolate activity, might contribute to the anti-acanthamoebic effects of *C. sinensis*.

*A. castellanii* secretes an abundant amount of serine protease and metalloprotease (Khan, 2006), which are known to compromise cell membrane integrity and cause cytolysis. In addition to being key determinants of the protozoan virulence (Dudley et al., 2008), these enzymes are also involved in the parasite differentiation into cysts (Alsam et al., 2005; Lorenzo-Morales et al., 2015). Interestingly, a study of *C. sinensis* polyphenols showed that EGCG appears to inhibit serine protease and metalloprotease (Benelli et al., 2002). Therefore, *C. sinensis* components (e.g. 3-O-gallate group) might interact with or even inhibit these enzymes, leading to cytolysis of *A. castellanii* trophozoites. Our TEM and SEM analyses showed ultrastructural intracellular and surface changes in *A. castellanii* exposed to *C. sinensis*. Of interest is the inhibition of the amoeba's ability to encyst, which is facilitated by cellulose, galactose and protein synthesis (Anwar et al., 2018). It is possible that some *C. sinensis* components may not only inhibit protease secretion, but also inhibit synthesis of other components, such as M17 leucine aminopeptidase of *A. castellanii* (AcLAP), which is involved and expressed in the late stages of encystation during the formation of the cyst wall (Lee et al., 2015).

## 5 Conclusion

We investigated the amoebicidal activity of *C. sinensis* against the trophozoite and cystic forms of *A. castellanii.* The results demonstrate that *C. sinensis* brews possess amoebicidal activity against *A. castellanii* trophozoites and were highly effective at inhibiting the parasite's ability to encyst*.* Also, *C. sinensis* interfered with the amoeba's ability to transform from cysts to trophozoites particularly at higher concentrations. These findings suggest that *C. sinensis* represents a source of potentially promising inhibitors of *A. castellanii* growth and encystation for the further development of topical complementary agents to support chemotherapeutic drugs currently used against *A. castellanii* infection. More studies are warranted to examine the anti-acanthamoebic activities of various solvent extracts of *C. sinensis* and various ingredients identified in the brewed green tea in this study. Also, the molecular mechanism of action of *C. sinensis* against *A. castellanii* is unknown and requires investigation. Additionally, it will be important to test the synergistic effect of *C. sinensis* and its constituents with different conventional anti-acanthamoebic drugs on the treatment of *A. castellanii* infection. The outcome of these studies could be helpful in the development of a safe, inexpensive and effective source of novel therapeutic agents against *A. castellanii* infections in the future.

## Authors’ contributions

Conceptualization: HME, CWS, XQZ. Formal analysis: LBF, HME. Methodology: LBF, HME. Project administration: HME, CWS. Resources: HME, XQZ. Supervision: HME, CWS. Writing of original draft: LBF. Writing - review & editing: HME, CWS, XQZ, LBF.

## Funding

Lenu Fakae is supported by a scholarship from the Nigerian Tertiary Education Trust Fund (10.13039/501100008895TETFund). Xing-Quan Zhu and Hany M. Elsheikha were supported by the International Science and Technology Cooperation Project of Gansu Provincial Key 10.13039/100006190Research and Development Program (Grant No. 17JR7WA031).

## Declaration of competing interest

The authors declare that they have no conflicts of interests.
