# Supplementary figures and images for: In vitro activity of Camellia sinensis (green tea) against trophozoites and cysts of Acanthamoeba castellanii
Source: Int J Parasitol Drugs Drug Resist. 2020 Jun 2;13:59–72. doi: 10.1016/j.ijpddr.2020.05.001 (PMC7281304; doi:10.1016/j.ijpddr.2020.05.001)

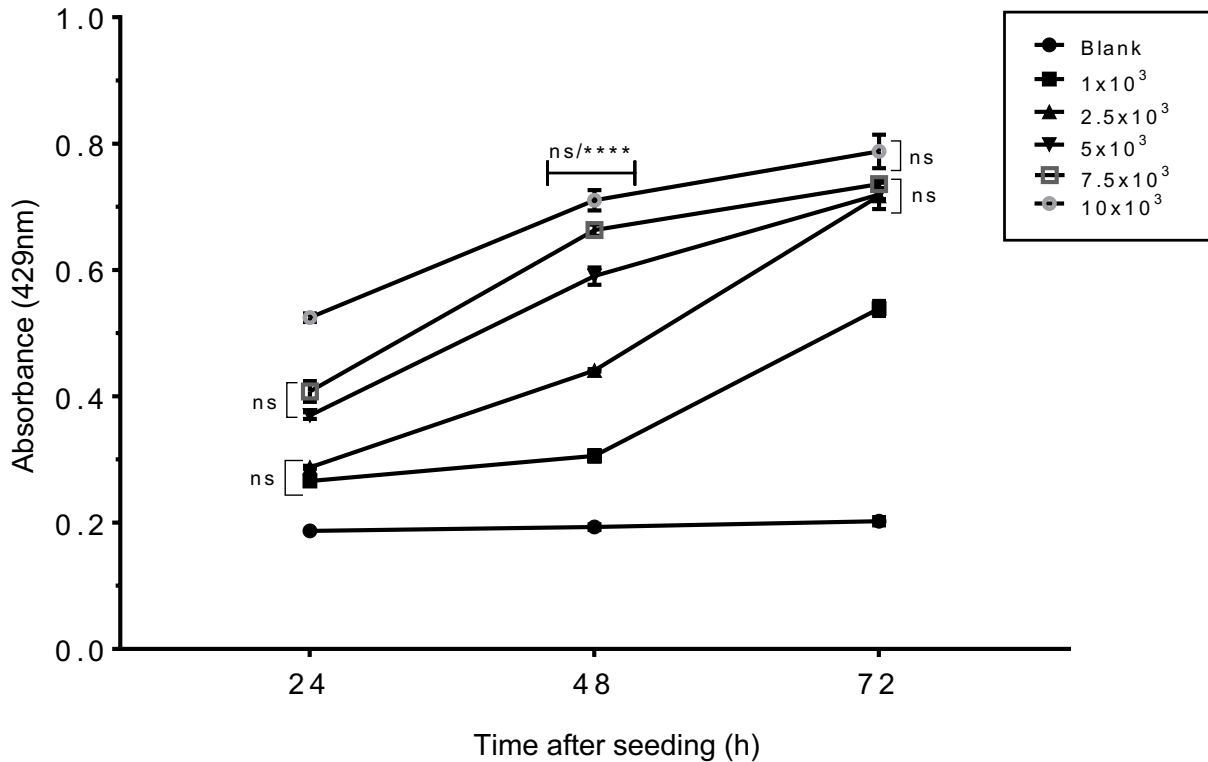

Supplement: Multimedia component 1 [file mmc1.pdf]

**24 h****48 h****72 h****Control**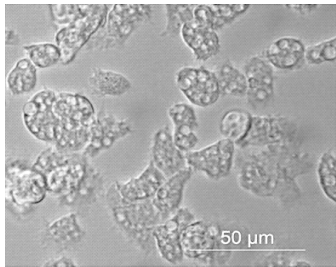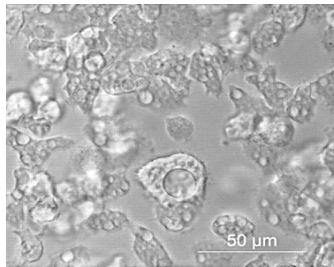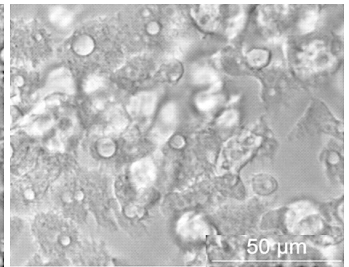**25%**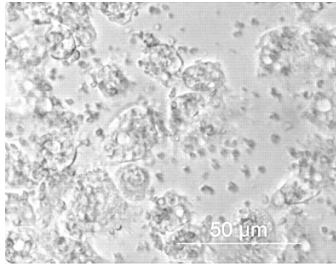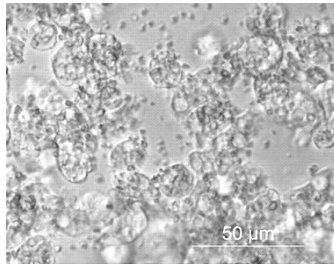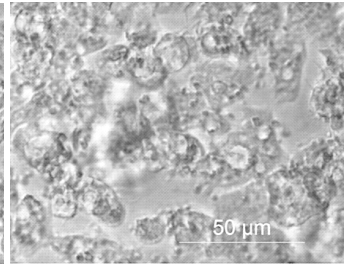**50%**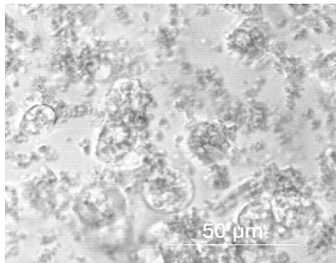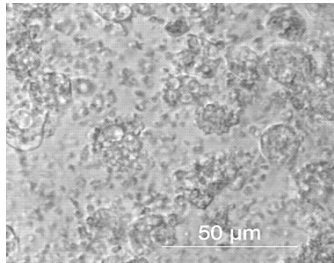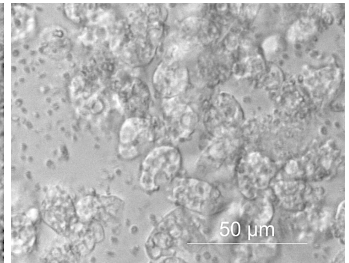**75%**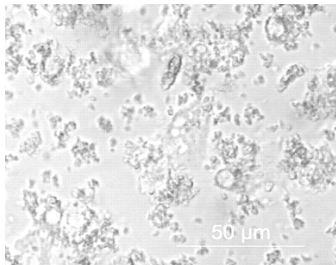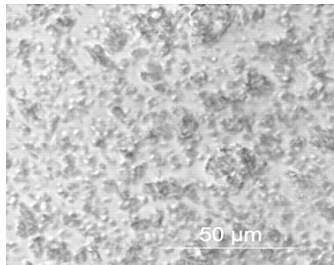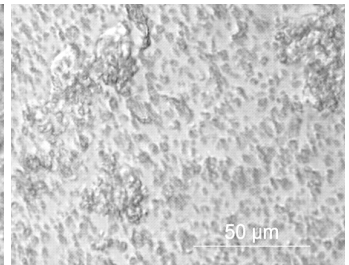**100%**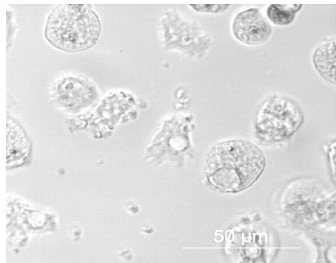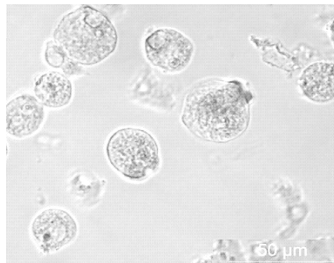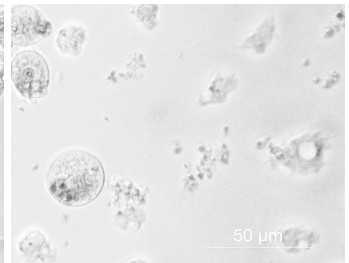

Supplement: Multimedia component 2 [file mmc2.pdf]

**12 h**

**48 h**

**72 h**

**Positive  
control**

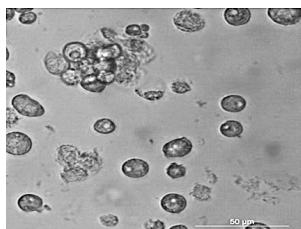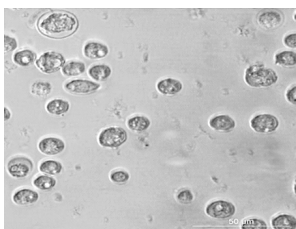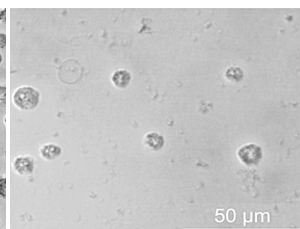

**Negative  
Control**

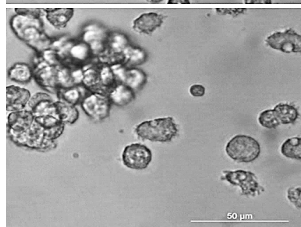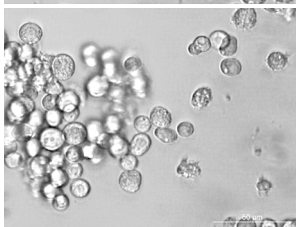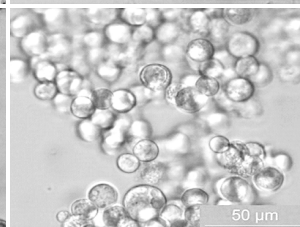

**25%**

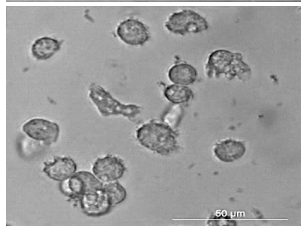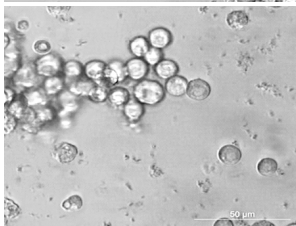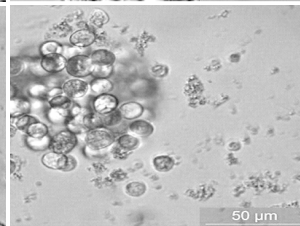

**50%**

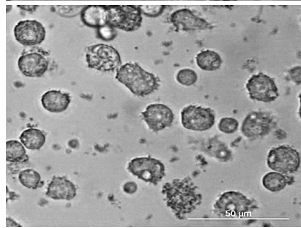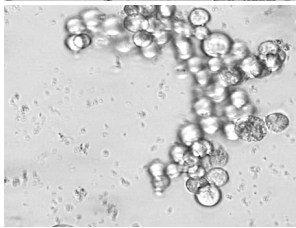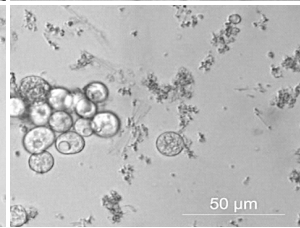

**75%**

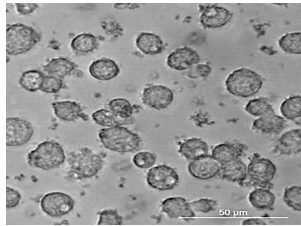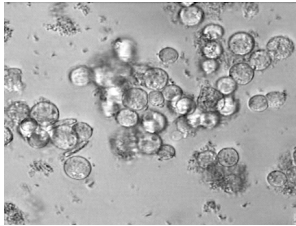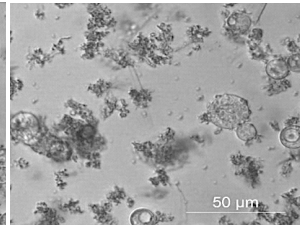

**100%**

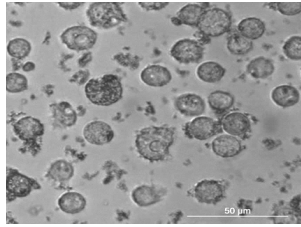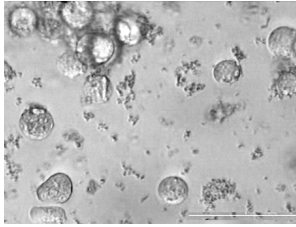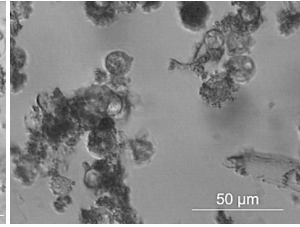

Supplement: Multimedia component 3 [file mmc3.pdf]

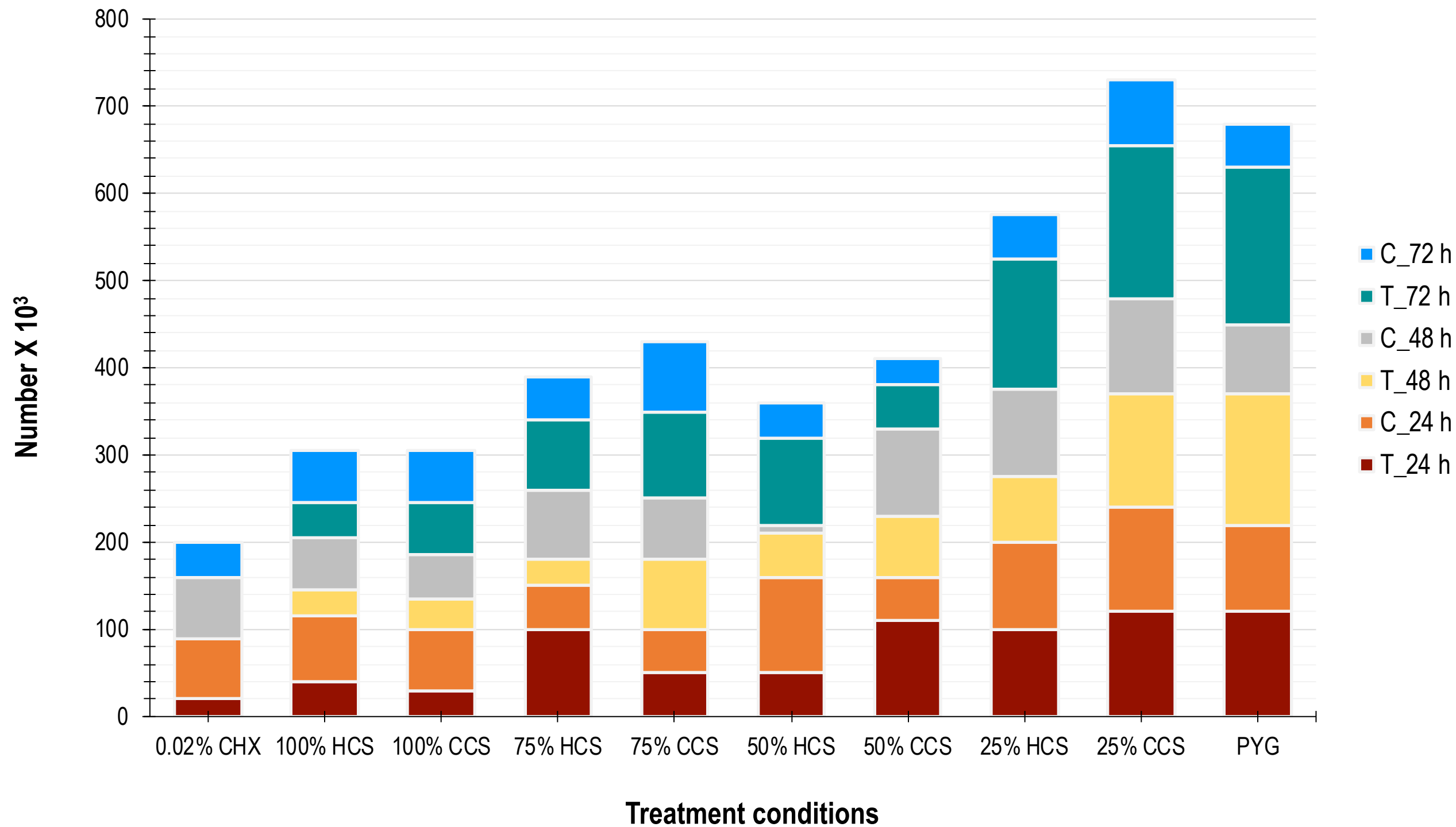

Supplement: Multimedia component 4 [file mmc4.pdf]
