# Supplementary material for: In vitro activity of Camellia sinensis (green tea) against trophozoites and cysts of Acanthamoeba castellanii
Source: Int J Parasitol Drugs Drug Resist. 2020 Jun 2;13:59–72. doi: 10.1016/j.ijpddr.2020.05.001 (PMC7281304; doi:10.1016/j.ijpddr.2020.05.001)
Supplement: Multimedia component 5 [file mmc5.docx]

**Supplemental figures:**

**Fig. S1.** Optimization of seeding density of *Acanthamoeba castellanii*. Trophozoites were seeded at the indicated numbers and their growth rate was determined at 24, 48 and 74 h using the SRB assay as described in materials and methods. No significant difference (ns) was detected between 7.5 × 10^3^ and 10 × 10^3^ at 48 h and 72 h (*p* > 0.05). **** *p* < 0.0001, ns *p* > 0.05.

**Fig. S2.** Morphological characteristics of *A. castellanii* trophozoites at 24, 48 and 72 h post-treatment with the indicated concentrations of hot *C. sinensis* brew*.* Control includes untreated *A. castellanii* trophozoites*.* Clear cellular destruction, inhibition of adherence to the flask surface, and excretions of cytoplasmic vesicles were observed in cultures treated with 25%, 50% and 75% of *C. sinensis*. These cellular changes increased in a time- and concentration-dependent manner. The cultures treated with 100% *C. sinensis* showed far less cytoplasmic vesicles excreted in the culture medium, however trophozoites became more rounded, less adherent to the surface of the flask and appeared as if they are trying to encyst. Scale bars = 50 µm.

**Fig. S3.** Morphological characteristics of *A. castellanii* at 12, 48 and 72 h post induction of encystation in the presence of *C. sinensis* brew at the indicated concentrations*.* Negative control includes *A. castellanii* trophozoites incubated in standardized encystation medium without *C. sinensis.* Positive control cultures were treated with 5mM PMSF. The rest of the images represent *A. castellanii* cultures treated with 25%, 50%, 75% and 100% hot *C. sinensis* encystation medium. A number of cellular fragments were observed at 12 h in the presence of 50%, 75% and 100% *C. sinensis*. Cellular destruction was observed at 48 h and by 72h increased cellular destruction was detected in the positive control and in *C. sinensis*-treated cultures in a concentration-dependent manner. Scale bar = 50 µm.

**Fig. S4**. Effect of *C. sinensis* on excystment of *A. castellanii* cysts. Cysts were incubated with 0.02% CHX, 25, 50, 75, and 100% hot *C. sinensis* brew (HCS) or cold *C. sinensis* brew (CCS). Control cysts were incubated in PYG medium. The numbers of cysts (C) and excysted trophozites (T) in samples exposed to the different indicated treatment conditions were counted at 24, 48 and 72 h post-exposure and displayed as a stacked-bar graph. Data represent the mean of three independent experiments performed in triplicate.
